# Fatty Liver/Adipose Tissue Dual‐Targeting Nanoparticles with Heme Oxygenase‐1 Inducer for Amelioration of Obesity, Obesity‐Induced Type 2 Diabetes, and Steatohepatitis

**DOI:** 10.1002/advs.202203286

**Published:** 2022-10-09

**Authors:** Juhyeong Hong, Yong‐Hee Kim

**Affiliations:** ^1^ Department of Bioengineering Institute for Bioengineering and Biopharmaceutical Research Hanyang University Seoul 04763 South Korea; ^2^ Education and Research Group for Biopharmaceutical Innovation Leader Hanyang University Seoul 04763 South Korea

**Keywords:** heme‐oxygenase‐1, nonalcoholic steatohepatitis, obesity‐induced metabolic syndrome, prohibitin targeting peptide, type 2 diabetes

## Abstract

Persistent uptake of high‐calorie diets induces the storage of excessive lipid in visceral adipose tissue. Lipids secreted from obese adipose tissue are accumulated in peripheral tissues such as the liver, pancreas, and muscle, and impair insulin sensitivity causing type 2 diabetes mellitus (T2DM). Furthermore, the accumulation of inflammatory cytokines and lipids in the liver induces apoptosis and fibrogenesis, and ultimately causes nonalcoholic steatohepatitis (NASH). To modulate obese tissue environments, it is challenged to selectively deliver inducers of heme oxygenase‐1 (HO‐1) to adipose tissue with the aid of a prohibitin targeting drug delivery system. Prohibitin binding peptide (PBP), an oligopeptide targeting prohibitin rich in adipose tissue, is conjugated on the surface of Hemin‐ or CoPP‐loaded poly(lactide‐*co*‐glycolide) nanoparticles (PBP‐NPs). PBP‐NPs efficiently differentiate lipid storing white adipocytes into energy‐generating brown adipocytes in T2DM and NASH models. In addition, PBP‐NPs are found to target prohibitin overexpressed fatty liver in the NASH model and inhibit hepatic uptake of circulating lipids. Furthermore, PBP‐NPs switch phenotypes of inflammatory macrophages in damaged organs and lower inflammation. Taken together, dual‐targeted induction of HO‐1 in fatty adipose and liver tissues is proven to be a promising therapeutic strategy to ameliorate obesity, insulin resistance, and steatohepatitis by lowering lipids and cytokines.

## Graphical abstract

Prohibitin binding peptide (PBP) conjugated nanoparticles (PBP‐NPs) efficiently induce heme oxygenase‐1 in adipocytes, hepatocytes, and tissue‐resident macrophages specifically in obesity‐related metabolic syndrome models. PBP‐NPs differentiate white adipocytes into brown adipocytes, boost hepatic mitochondrial biogenesis, and polarize macrophages into M2 types inhibiting inflammation and fibrogenesis. Fatty liver/adipose tissue dual‐targeted synergistic effects through clearing lipids and cytokines are demonstrated in detail.

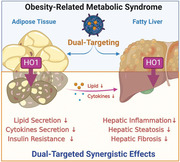

## 1 Introduction

Metabolic syndromes are multicomponent disorders associated with visceral obesity, dyslipidemia, impaired insulin sensitivity, and chronic inflammation.^[^ ^1^ ^]^ In obesity, high calorie‐diets uptake induces the accumulation of excessive lipid droplets in adipose tissue.^[^ ^2^, ^3^ ^]^ Excessive lipid in adipose tissue by high‐saturated‐fat diets increases levels of nonesterified fatty acids (NEFAs) also known as free fatty acids (FFAs) in serum, which induce triglyceride (TG) storage in peripheral tissues.^[^ ^4^, ^5^ ^]^ These result in an imbalance of lipid composition with high levels of saturated NEFA and lower levels of polyunsaturated fatty acids regulating receptors related to glucose transport and insulin sensitivity.^[^ ^6^, ^7^ ^]^ In addition, obese adipose tissues secrete several cytokines causing a chronic inflammatory state and eventually lead to metabolic failure like type 2 diabetes mellitus (T2DM).^[^ ^8^, ^9^ ^]^ Furthermore, excessive lipids induce liver abnormalities, known as nonalcoholic fatty liver disease, characterized by an increase in TG contents in the liver.^[^ ^10^, ^11^ ^]^ Progress of overloaded TG to hepatocyte mitochondrial dysfunction, reactive oxygen species (ROS) production, and hepatic inflammation and fibrogenesis eventually cause the nonalcoholic steatohepatitis (NASH).^[^ ^11^, ^12^, ^13^, ^14^ ^]^ Therefore, inhibiting the secretion of lipids and cytokines from obese adipose tissue following lipid storage would be an effective therapeutic strategy for obesity and obesity‐induced metabolic syndromes.

Heme oxygenase‐1 (HO‐1) catalyzes the breakdown of heme into biliverdin and carbon monoxide with a concomitant release of iron.^[^ ^15^, ^16^ ^]^ Furthermore, HO‐1 provides a protective response to ROS or stress associated with inflammation.^[^ ^17^ ^]^ In recent studies, HO‐1 upregulation in adipocytes was reported to attenuate lipid accumulation in obese mice by differentiating white adipose tissue into brown adipose tissue, a phenomenon known as brown adipogenesis.^[^ ^18^, ^19^ ^]^ Differentiated brown adipocytes stimulate mitochondrial biogenesis and facilitate thermogenesis via fatty acid beta‐oxidation by exhausting free fatty acids.^[^ ^20^, ^21^, ^22^ ^]^ In addition, HO‐1 upregulation increased adiponectin levels and insulin sensitivity in obese mice and provided evidence for the amelioration of the chronic inflammatory state that results in metabolic syndromes like T2DM and NASH.^[^ ^21^, ^23^, ^24^, ^25^, ^26^ ^]^ HO‐1 was also proven to modulate phenotypes of tissue‐resident macrophages and decrease levels of tumor necrosis factor‐*α* (TNF‐*α*) and interleukin‐6 (IL‐6) in inflammatory diseases.^[^ ^27^ ^]^ The induction of HO‐1 could protect tissues against M1 macrophage‐mediated inflammatory responses by preferentially provoking the anti‐inflammatory M2 phenotype.^[^ ^28^ ^]^ For these potential therapeutic effects, recent studies have focused on the induction of HO‐1 in the injured organs of several acute or chronic inflammatory diseases.^[^ ^29^, ^30^, ^31^ ^]^ However, as obese adipose tissue environments are hard to modulate as overloaded lipids and cytokines cause chronic inflammation, sustainable high induction of HO‐1 compared to short term expression is required to overcome obesity‐induced metabolic syndrome.^[^ ^25^ ^]^

Nuclear factor erythroid 2‐related factor 2/heme oxygenase 1 (Nrf2/HO‐1) signaling axis has been widely studied for its effective therapeutic effects in several inflammatory diseases.^[^ ^32^, ^33^, ^34^, ^35^ ^]^ However, Nrf2, a leucine zipper transcriptional activating factor cap‐n‐collar (CNC) family, is regulated by kelch‑like ECH‑associated protein 1 (KEAP1) chaperone via degrading it through ubiquitination and proteasome‐dependent degradation.^[^ ^36^, ^37^ ^]^ Consequently, to highly stimulate HO‐1 activity in cells, blocking KEAP1 from degrading Nrf2 is an effective approach. Iron protoporphyrin ix (hemin) and cobalt protoporphyrin ix (CoPP) are tetrapyrrole drugs containing iron or cobalt which strongly activate intracellular redox activity.^[^ ^38^ ^]^ Strongly activated intracellular redox activity occurred by hemin or CoPP reacts with critical cysteine residues of KEAP1, leads to Nrf2 dissociation from KEAP1 and nuclear translocation.^[^ ^39^ ^]^ Consequently, Nrf2 coordinates with Maf protein and binds to the antioxidant response element to express the gene of HO‐1.^[^ ^37^ ^]^ Thus, hemin and CoPP could strongly induce HO‐1 mRNA transcriptions and showed great anti‐inflammatory therapeutic effects long enough in several studies.

We developed HO‐1 inducing nanoparticles targeting adipose tissue comprised of white adipocytes and adipose tissue‐derived macrophages, by modifying poly(lactide‐*co*‐glycolide) (PLGA) nanoparticles with the prohibitin binding peptide (PBP, CKGGRAKDC) which was confirmed to target prohibitin located on the cell surface.^[^ ^40^ ^]^ Prohibitin is highly expressed in the inner mitochondrial membrane and shifts its location when lipid storing white adipocytes are fully differentiated.^[^ ^40^, ^41^ ^]^ In our previous studies, various types of genetic material were specifically delivered to mature adipocytes with high efficiency in vitro and in vivo using a prohibitin‐mediated drug delivery system.^[^ ^40^, ^42^, ^43^, ^44^ ^]^ In the current study, we manufactured PBP‐conjugated HO‐1 inducer‐loaded nanoparticles (PBP‐NPs) and analyzed physicochemical characteristics. To understand the targeting ability and therapeutic effects of PBP‐NPs, we utilized murine adipocytes, macrophages, and their co‐culture system in vitro. In addition, the efficacy of inhibiting the secretion of lipids and cytokines from obese adipose tissue by PBP‐NPs was examined in the type 2 diabetes mellitus mouse model and NASH mouse model. It was found that PBP‐NPs modulated environments of obese adipose tissue and ameliorated disease pathology. Furthermore, we demonstrated the overexpression of prohibitin in the fatty liver and PBP‐mediated drug delivery systems could upregulate HO‐1 in the injured liver for antiapoptosis, anti‐inflammation, and hepatic macrophage phenotype switching.

## 2 Results

### 2.1 Prohibitin Binding Peptide (PBP)‐Conjugated Nanoparticles for Heme Oxygenase‐1 (HO‐1) Inducer Delivery

**Figure 1:**
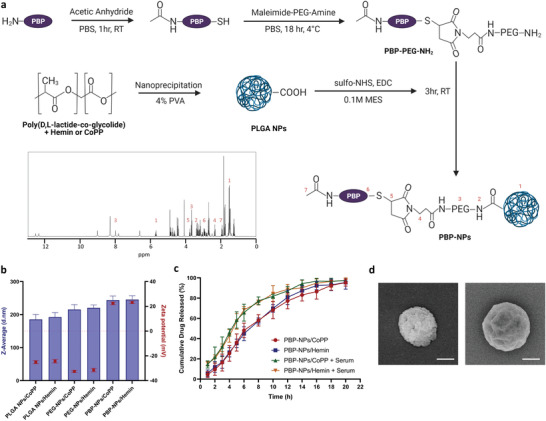
Preparation and characterization of prohibitin binding peptide (PBP)‐conjugated PLGA nanoparticles (PBP‐NPs) containing heme oxygenase‐1 (HO‐1) inducers, Hemin and CoPP. a) Hemin or CoPP was encapsulated in the PLGA nanoparticles by dissolving them in dichloromethane solution and dropping organic solution into aqueous solution with 4% under stirring. After nanoprecipitation, PLGA NPs were sonicated and centrifuged to isolate PLGA NPs. To conjugate PBP on PLGA NPs, maleimide‐PEG (2000)‐amine was used as a linker between PLGA NPs and PBP peptides. The blocking N‐terminal end of the peptide was performed with acetic anhydride to prevent direct conjugation with PLGA NPs during sulfo‐NHS/EDC mediated conjugation. Modified peptides followed dialysis to conjugate with maleimide‐PEG (2000)‐amine forming a thioether bond with the thiol group of cysteine in PBP. PBP‐PEG‐NH2 were purified using size‐exclusion chromatography and dissolved in 0.1 m MES buffer with PLGA NPs to initiate EDC/sulfo‐NHS covalent couplings. The figure was created with BioRender.com. Peaks of each active group are marked in the H NMR spectrum of the PBP‐NPs. b) Z‐average diameter and zeta potential of PLGA NPs were measured using dynamic light scattering (*n* = 12). c) Drug release kinetics were measured in PBS with or without serum for 22 h by using a UV‐vis spectrophotometer (*n* = 12). d) Representative SEM images of PLGA NPs (left) and PBP‐NPs (right). Scale bar = 100 nm. Data are presented as means ± SD.

HO‐1 inducers, hemin and cobalt protoporphyrin ix (CoPP), were encapsulated in the PLGA nanoparticles (PLGA NPs) via nanoprecipitation by dropping the organic phase to aqueous phase with 4% poly(vinyl alcohol) (PVA). To conjugate a targeting moiety to PLGA NPs, maleimide‐PEG (2000)‐amine was used as a linker between PLGA NPs and PBP. Blocking the N‐terminal end of the peptide was performed with acetic anhydride to prevent direct conjugation with PLGA NPs during *N*‐hydroxysulfosuccinimide sodium (sulfo‐NHS)/*N*‐(3‐di methyl aminopropyl)‐*N*′‐ethyl carbodiimide hydrochloride (EDC) mediated conjugation. Modified peptides followed dialysis to conjugate with maleimide‐PEG (2000)‐amine forming a thioether bond with the thiol group of cysteine in PBP. PBP‐PEG‐NH2 was further purified using size‐exclusion chromatography and dissolved in 0.1 m MES (2‐(*N*‐morpholino)ethanesulfonic acid) buffer with PLGA NPs to initiate EDC/sulfo‐NHS covalent coupling. Finally, PBP‐NPs containing hemin or CoPP were lyophilized for further experiments. The schematic illustration of PBP‐NPs preparation is shown below (**Figure** **1**a).

The chemical conjugation on the final nanoparticles was analyzed by H NMR (Figure 1a). The EDC/sulfo‐NHS reaction (between polyethylene glycol (PEG) and PLGA) was proved by the presence of 3.37 (amide bond), 3.67 (–CH2 in PEG), 4.49 (–CH2 in PLGA), and 5.71 (–CH in PLGA) peaks at H NMR. Also, the maleimide‐thiol reaction was proved by the presence of 2.81–3.06 (between cysteine and maleimide) peaks at H NMR. Mean particle sizes of PLGA NPs were 188.9 ± 14.5 nm and the sizes of PBP‐NPs containing drugs increased to 244.7 ± 12.1 nm on average (Figure 1b). In addition, the zeta potential of PBP‐NPs showed a positive charge of around 25 mV, suggesting that conjugation of PBP reversed the surface charge of nanoparticles (Figure 1b). To calculate the encapsulation efficiency and drug loading, PBP‐NPs were dissolved in dichloromethane (DCM)/dimethylsulfoxide (DMSO)/acetonitrile (ACN) solution and analyzed with a UV‐vis spectrophotometer with the standard curve for each HO‐1 inducer as described in the Experimental Section. The encapsulation efficiency and drug loading of hemin‐loaded PBP‐NPs (PBP‐NPs/Hemin) were 68 ± 5.6% and 5.4 ± 1.5%, and CoPP‐loaded PBP‐NPs (PBP‐NPs/CoPP) were 64 ± 4.2% and 5.8 ± 0.8%, respectively. Drug release profiles showed sustained release patterns without initial bursts. Around 50% of total amounts of HO‐1 inducers were released at 6 h and the remaining HO‐1 inducers were continuously released up to 100% until 22 h, and around 10% more were released in the presence of serum (Figure 1c). Finally, PBP‐NPs showed larger diameters and changes in the surface in scanning electron microscopy (SEM) image (Figure 1d)

### 2.2 PBP‐NPs Promote Brown Adipogenesis by HO‐1 Overexpression

**Figure 2:**
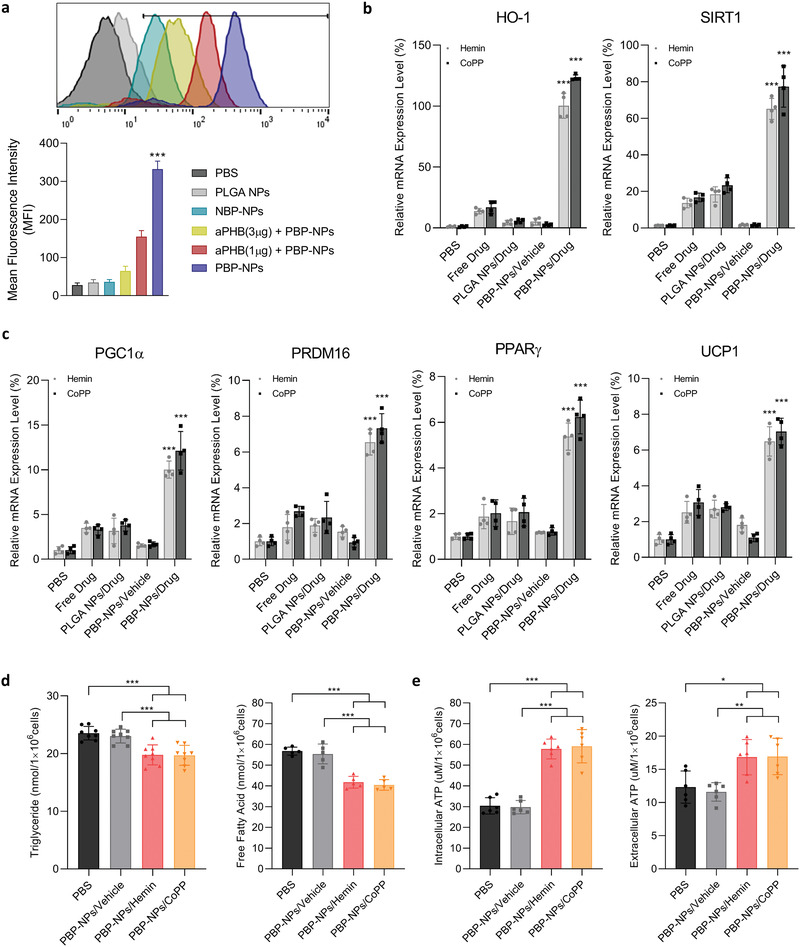
Selective uptake, HO‐1 upregulation, and brown adipogenesis of PBP‐NPs in 3T3‐L1 cells. a) Prohibitin‐mediated cellular uptake of PBP‐NPs was analyzed by flow cytometry following 1 µg (red) and 3 µg (yellow) of antiprohibitin antibody (aPHB) preincubation for 2 h. Nonbinding peptide, NBP (CGGRGAGAC), was compared as a negative control (*n* = 4). b) mRNA levels of HO‐1 and SIRT1 in 3T3‐L1 cells (*n* = 4). c) mRNA levels of brown adipogenesis (PGC1*α*, PRDM16, PPAR*γ*, and UCP1) in 3T3‐L1 cells (*n* = 4). d) Intracellular triglyceride (*n* = 8) and free fatty acid (n = 5) levels after 48 h treatments, representing lipid burning effects of brown adipocytes. e) Intracellular and extracellular ATP levels resulting from fatty acid beta‐oxidation of brown adipocytes (*n* = 6). Data are presented as means ± SD. ns = not significant, *p < 0.033, **p < 0.01, ***p < 0.001, by one‐way ANOVA with Tukey's post hoc test were considered.

In order to verify the mature adipocyte targetability of PBP‐NPs, Cy5.5 dye‐encapsulated PBP‐NPs were treated with mature adipocytes differentiated from 3T3‐L1 cells and compared with PLGA NPs, nonbinding peptide (NBP, scrambled peptide)‐modified NPs (NBP‐NPs), and receptor blocking antibody (aPHB) pretreated groups. PBP‐NPs were found to accumulate in adipocytes around six times higher than NBP‐NPs and as expected, preincubation of mature adipocytes with antiprohibitin antibody (aPHB) which competes with PBP on prohibitin binding significantly reduced cellular uptake of PBP‐NPs in a dose‐dependent manner (**Figure** **2**a and Figure S1, Supporting Information). These results represent that PBP‐NPs are able to target prohibitin on the cell surface and deliver cargos selectively into white adipocytes.

The toxicity of HO‐1 inducer‐loaded PBP‐NPs was evaluated in a dose‐dependent manner. Cell viability was observed to remain above 80% up to 200 × 10^−6^ m of hemin‐loaded PBP‐NPs and CoPP‐loaded PBP‐NPs in mature adipocytes (Figure S2, Supporting Information). In addition, to verify the effectiveness of HO‐1 inducer‐loaded PBP‐NPs, mature adipocytes were treated with PBP‐NPs/Hemin and PBP‐NPs/CoPP and intracellular HO‐1 mRNA levels were measured. Much higher expression of HO‐1 was obtained in the PBP‐NPs group compared with free drugs, PLGA NPs containing drugs, and PBP‐NPs not containing drugs, which represents the prohibitin‐targeted delivery of HO‐1 inducer with PBP‐NPs was highly and selectively efficient in white adipocytes (Figure 2b).

Furthermore, overexpression of HO‐1 induced upregulation of Sirtuin‐1 (SIRT1), NAD+‐dependent deacetylases, which is related to cellular processes including energy metabolism (Figure 2b). Overexpressed SIRT1 leads to the deacetylation and activation of peroxisome proliferator‐activated receptor‐gamma (PPAR*γ*) related to transcriptional regulation of brown adipogenesis (Figure 2c). In addition, PPAR*γ* coactivator 1*α* (PGC1*α*) and PR domain‐containing protein 16 (PRDM16), which are key regulators of brown adipocyte‐specific genes such as uncoupling protein‐1 (UCP1), were found to be highly activated in PBP‐NPs/Hemin and PBP‐NPs/CoPP groups (Figure 2c). Consequently, expression of uncoupling protein‐1 (UCP1) in the inner mitochondrial membrane, which allows dissipation of the proton electrochemical gradient generated by respiration in the form of heat, was highly induced (Figure 2c). The activation of the brown adipogenic/thermogenic transcriptional cascade resulted in the reduction of accumulation of free fatty acids and triglyceride levels in adipocytes (Figure 2d). These results are due to the enhanced lipid burning to generate abundant ATPs, which is a characteristic functional property of brown adipocytes (Figure 2e).

### 2.3 PBP‐NPs Promote Phenotype Switching of Adipose Tissue‐Derived Macrophages into Anti‐Inflammatory M2 Types

**Figure 3:**
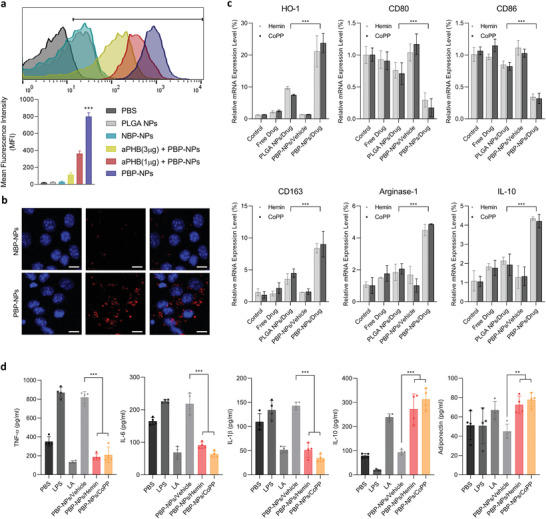
Phenotype switching of adipose tissue‐derived macrophages by PBP‐NPs in macrophages. a) Prohibitin‐mediated cellular uptake of PBP‐NPs was analyzed by flow cytometry following 1 µg (red) and 3 µg (yellow) of antiprohibitin antibody (aPHB) preincubation for 2 h. Nonbinding peptide, NBP (CGGRGAGAC), was compared as a negative control (*n* = 4). b) Comparison of cellular uptake between Cy5.5‐loaded PBP‐NPs and NBP‐NPs following 24 h treatments. Nucleus (blue) and Cy5.5 (red) were visualized. Scale bar = 20 µm. c) mRNA levels of HO‐1, inflammatory M1 macrophage markers (CD80 and CD86), and anti‐inflammatory M2 macrophage markers (CD163, Arginase‐1, and IL‐10) (*n* = 4). d) Raw 264.7 cells were preincubated with lipopolysaccharide (LPS, M1 phenotype inducer) for 2 h. After activation, lactic acid (LA, M2 phenotype inducer) or PBP‐NPs were treated for 24 h. Then macrophages were seeded in a plate where mature 3T3‐L1 cells were already cultured. Media were collected after 48 h of co‐culture and cytokine levels were analyzed by ELISA (*n* = 4). Data are presented as means ± SD. ns = not significant, *p < 0.033, **p < 0.01, ***p < 0.001, by one‐way ANOVA with Tukey's post hoc test were considered.

Our previous study has shown that visceral adipose tissue‐derived macrophages (ATMs) causing obesity‐induced inflammation by releasing inflammatory cytokines such as tumor necrosis factor‐alpha (TNF‐*α*) and interleukin‐6 (IL‐6) overexpress prohibitin on its surface.^[^ ^42^ ^]^ Also, it has been reported that HO‐1 upregulation in macrophages ameliorates inflammation and switches its phenotype into anti‐inflammatory M2 types.^[^ ^28^ ^]^ ATMs were isolated from visceral adipose tissue of high‐fat diet‐induced type 2 diabetic mice. Cy5.5‐loaded PBP‐NPs were found to be selectively uptaken by ATM via prohibitin binding and pretreatment with aPHB reduced uptake of PBP‐NPs into ATM in a dose‐dependent manner (**Figure** **3**a). In addition, confocal laser scanning microscopy images showed high localization of PBP‐NPs inside ATM even after 24 h, while the Cy5.5 signal almost vanished in the NBP‐NPs group (Figure 3b). The HO‐1 inducer‐loaded PBP‐NPs significantly upregulated HO‐1 mRNA levels compared to other groups and consequently, phenotypes of ATM were switched to anti‐inflammatory M2 types without cytotoxicity (Figure 3c and Figure S3, Supporting Information). The mRNA levels of inflammatory ATM markers (CD80 and CD86) were significantly decreased by more than 70% in both PBP‐NPs/Hemin and PBP‐NPs/CoPP groups and mRNA levels of anti‐inflammatory M2 macrophage markers (CD163, Arginase‐1, and IL‐10) were proportionally increased (Figure 3c).

ATM and adipocytes have been known to crosstalk and differentiate to induce inflammation in obese adipose tissue, which develops into obesity‐induced metabolic syndrome.^[^ ^45^ ^]^ To evaluate the dual targeting effects of PBP‐NPs on ATM and adipocytes, the direct co‐culture system mimicking adipose tissue environment was used. Murine monocyte cells (RAW 264.7) were activated by lipopolysaccharide (LPS) to provoke inflammation, followed by treatment with lactic acid and PBP‐NPs for 6 h to stimulate phenotype switching. Then macrophages were transferred to the mature 3T3‐L1 plates to confirm the reduced inflammation by phenotype‐switched M2 anti‐inflammatory macrophages. High levels of inflammatory cytokines such as interleukin‐6 (IL‐6), interleukin‐1beta (IL‐1*β*), and tumor necrosis factor‐alpha (TNF‐*α*) in culture media by LPS activation were significantly decreased by treatment with HO‐1 inducer‐loaded PBP‐NPs, which indicates that phenotype‐switched macrophages altered the environment against inflammation (Figure 3d).

### 2.4 Targeted Delivery of PBP‐NPs to White Adipose Tissue Induces Obesity Reduction, Brown Adipogenesis, and Macrophage Phenotype Switching in High‐Fat Diet‐Induced Type 2 Diabetic Mice

HO‐1 inducer‐loaded PBP‐NPs could effectively differentiate white adipocytes into brown adipocytes which exhaust lipids and decrease inflammation by switching macrophages into anti‐inflammatory M2 types. Therefore, HO‐1 inducer‐loaded PBP‐NPs were considered to be novel therapeutics for obesity‐induced metabolic syndromes such as T2DM and NASH occurred by adipose tissue dysfunction with excessive NEFA, inflammation, and oxidative stress.

**Figure 4:**
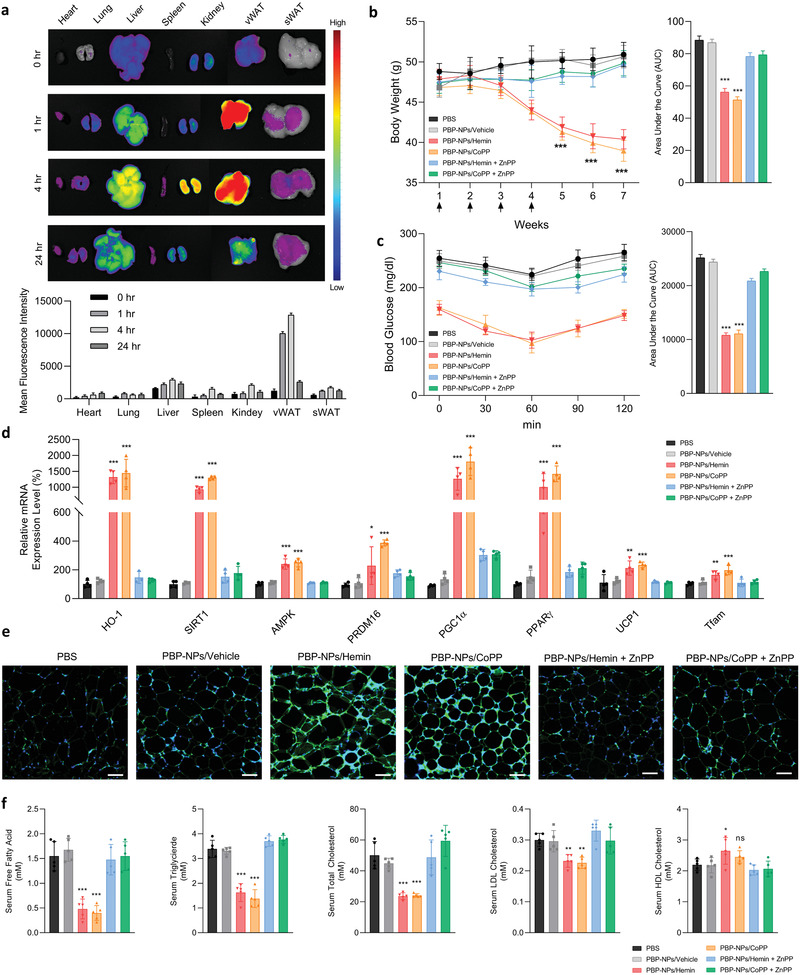
Adipose tissue‐targeting and therapeutic effects of PBP‐NPs in high‐fat diet‐induced type 2 diabetic mouse model. a) Ex vivo biodistribution image of intraperitoneally injected Cy 5.5‐loaded PBP‐NPs and mean fluorescence intensity per tissue area of major organs (*n* = 3) NPs in high‐fat diet‐induced type 2 diabetic mouse model. b) Measurements of body weight change. The high‐fat diet‐induced type 2 diabetic mouse model was intravenously injected with 1 mg kg^−1^ of nanoparticles once a week for 4 weeks and ZnPP, an HO‐1 inhibitor, was intraperitoneally injected to study competitive inhibition with PBP‐NPs containing HO‐1 inducers (*n* = 5). c) Insulin tolerance tests were conducted following intraperitoneal injection of insulin into fasted mice post 7 weeks of treatments. d) Relative mRNA expression levels of HO‐1, downstream signals (SIRT1 and AMPK), brown adipogenesis markers (PRDM16, PPAR*γ*, and PGC1*α*), and mitochondrial biogenesis markers (UCP1 and Tfam) in visceral adipose tissue. The mRNA levels were normalized to that of GAPDH mRNA by qPCR (*n* = 4). e) Immunofluorescence staining of UCP1 (green) in visceral adipose tissue embedded in paraffin. Section thickness = 6 µm. Scale bar = 200 µm. f) Serum levels of free fatty acids, triglyceride, total cholesterol, LDL cholesterol, and HDL cholesterol (*n* = 5). Data are presented as means ± SD. ns = not significant, *p < 0.033, **p < 0.01, ***p < 0.001, by one‐way ANOVA with Tukey's post hoc test were considered.

An obesity‐induced type 2 diabetic mouse model was prepared by feeding 60 kcal% high‐fat diets for 12 weeks to 6 weeks old male C57BL/6J as we reported before.^[^ ^40^, ^42^, ^43^, ^44^ ^]^ A NASH mouse model was prepared by feeding 60 kcal% high‐fat diets followed by tap water with 25% fructose for 28 weeks to 6 weeks old male C57BL/6J as described in the Experimental Section. Cy5.5‐loaded PBP‐NPs were injected intravenously into a high‐fat diet‐induced type 2 diabetic mouse model to confirm the accumulation of PBP‐NPs into visceral white adipose tissue (vWAT) where prohibitin expression was significantly higher than subcutaneous white adipose tissue (sWAT). After 1 h of injection, PBP‐NPs were localized in vWAT and the signal was maintained until 24 h (**Figure** **4**a). Based on the vWAT targetability of PBP‐NPs, two types of HO‐1 inducer, hemin and CoPP, were encapsulated in PBP‐NPs and antiobesity and antidiabetes effects of resulting targeted HO‐1 overexpression were investigated. The high‐fat diet‐induced type 2 diabetic mouse model was intravenously injected with 1 mg kg^−1^ of nanoparticles once a week for 4 weeks and ZnPP, an HO‐1 inhibitor, was intraperitoneally injected to study competitive inhibition with PBP‐NPs containing HO‐1 inducers. PBP‐NPs/Hemin or PBP‐NPs/CoPP‐injected groups demonstrated remarkable weight loss of about 20% and the effects were maintained even after stopping injection until 7 weeks (Figure 4b and Figures S4 and S5, Supporting Information). We also measured weekly food uptake to see whether the treatment affects calorie intake and causes weight loss, but there was no difference for all groups (Figure S6, Supporting Information). After 7 weeks of treatments, an insulin tolerance test was performed with 6 h fasted mice. PBP‐NPs efficiently lowered fasted blood glucose levels to normal levels contrary to other groups, which represented a recovery of insulin resistance (Figure 4c).

Furthermore, we isolated mRNAs of vWAT tissues from treated mice and analyzed downstream signaling of HO‐1. Downstream signals, SIRT1 and AMPK, stimulate the thermogenic gene program in white adipocytes via PGC‐1*α* activation with its cofactors such as PRDM16 and PPAR. We confirmed that HO‐1 overexpression by PBP‐NPs increased SIRT1 and AMPK levels, and consequently increased brown adipogenesis markers (PRDM16, PPAR*γ*, and PGC1*α*) in vWAT (Figure 4d). In addition, mitochondrial transcription factor A (Tfam) known as a mitochondrial biogenesis marker was highly increased, which represented that adipose tissue produced energy via fatty acid oxidation in mitochondria (Figure 4d). Furthermore, the immunofluorescence image of mitochondrial biogenesis and brown adipogenesis marker, UCP1, demonstrated that HO‐1 overexpression led to the differentiation of adipocytes into energy‐producing types (Figure 4e). High protein levels of translocase of outer membrane of mitochondria (TOM20) and brown adipogenesis markers (PGC1*α* and UCP1) in adipose tissues were confirmed with western blot, and brown adipocyte‐like morphological alternations of adipose tissue were shown in hematoxylin and eosin (H&E) staining (Figure S7, Supporting Information). Thermogenic activation via PBP‐NPs containing HO‐1 inducers decreased levels of free fatty acids in serum from the third week of treatment and the effects were boosted until the seventh week (Figure S8, Supporting Information). Consequently, lowered free fatty acids, triglyceride, cholesterol, and low‐density lipoprotein (LDL) levels in plasma were observed in HO‐1‐induced groups and high‐density lipoprotein (HDL) was increased (Figure 4f). Taken together, targeted HO‐1 overexpression in white adipose tissue activated brown adipogenesis and mitochondrial biogenesis, and burned FFAs by turning them into energy after the brown adipocytes portion increased after the third week.

**Figure 5:**
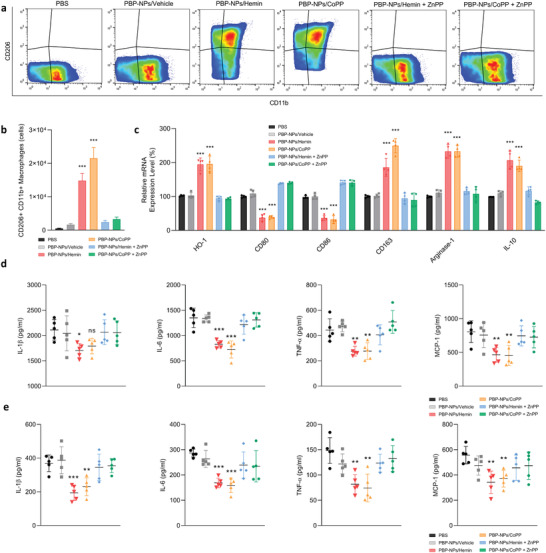
Macrophage phenotype switching and reduction of inflammatory cytokines by PBP‐NPs in high‐fat diet‐induced type 2 diabetic mouse model. a) Phenotypes of adipose tissue macrophage isolated from visceral adipose tissue were analyzed by flow cytometry (*n* = 3). b) CD206+CD11b+ M2 macrophages were quantified (*n* = 3). c) Relative mRNA levels of SVF from visceral adipose tissue. The mRNA levels were normalized to that of GAPDH mRNA by qPCR (*n* = 4). d) Inflammatory cytokines in visceral adipose tissue were analyzed by ELISA (*n* = 5). e) Inflammatory cytokines in serum were analyzed by ELISA (*n* = 5). Data are presented as means ± SD. ns = not significant, *p < 0.033, **p < 0.01, ***p < 0.001, by one‐way ANOVA with Tukey's post hoc test were considered.

Immune cells near fatty adipose tissues are highly related to obesity‐induced metabolic syndrome via cytokine secretion and apoptosis induction.^[^ ^46^ ^]^ Total adipose tissue‐derived macrophages were isolated from vWAT after PBP‐NPs treatment to identify the macrophage population. The CD11b+ CD206+ macrophages which represent anti‐inflammatory M2 types were found to be abundant in PBP‐NPs‐treated groups, while other groups showed quite fewer populations (**Figure** **5**a,b). Extracted RNAs from isolated macrophages were transformed into cDNAs and relative gene expression levels were analyzed by quantitative polymerase chain reaction (qPCR). PBP‐NPs‐treated groups showed high HO‐1 expression. Also, while inflammatory M1 macrophage markers (CD80 and CD86) were significantly downregulated, M2 macrophage markers (CD163, Arginase‐1, and IL‐10) were more than doubled than other groups (Figure 5c). In addition, levels of proinflammatory cytokines in tissue lysate and serum were noticeably decreased in the HO‐1 induced groups (Figure 5d,e). Finally, the crown‐like structures caused by infiltrated M1 macrophages in obese adipose tissues were diminished, which could reduce the apoptosis of adipocytes (Figure S9, Supporting Information). Taken together, adipose tissue‐derived cytokines causing insulin resistance were proven to be significantly diminished in mouse models via phenotype switching of ATM by PBP‐NPs containing HO‐1 inducers.

### 2.5 Fatty Liver‐Targeting and Therapeutic Effects of PBP‐NPs in a High‐Fat/High‐Fructose Diet‐Induced NASH Mouse Model

NASH is one of the most common chronic liver diseases, which is highly associated with obesity‐induced metabolic syndrome and caused by high lipid accumulation in the vWAT and liver.^[^ ^5^, ^47^, ^48^ ^]^ However, none of the drugs have been approved by the Food and Drug Administration (FDA) yet for the complexity of NASH pathophysiology. Highly accumulated lipids in the liver induce hepatic ballooning and apoptosis, and cytokines have been known to be the main triggers for hepatic macrophages to induce inflammation and activation of stellate cells causing fibrosis in the liver.^[^ ^47^, ^49^ ^]^ Consequently, the reduction of lipids and inflammatory cytokines accumulated in the fatty liver is a promising therapeutic strategy for the treatment and prevention of NASH.

**Figure 6:**
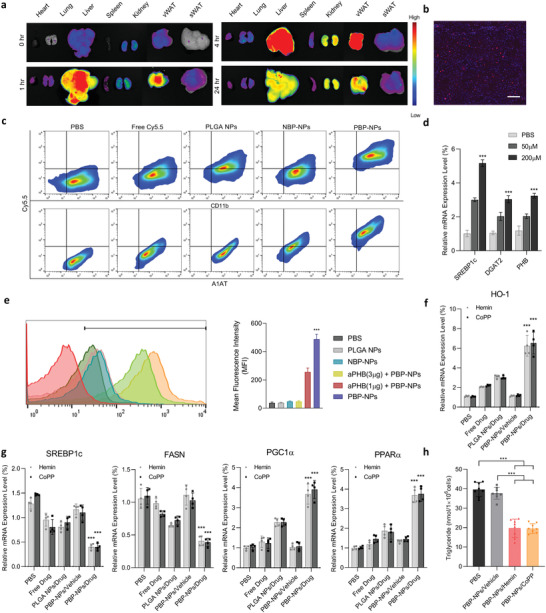
Fatty liver‐targeting and normalization of lipid metabolism of PBP‐NPs in a high‐fat/high‐fructose diet‐induced NASH mouse model. a) Ex vivo biodistribution image of intravenously injected Cy5.5‐loaded PBP‐NPs in a high‐fat/high‐fructose diet‐induced NASH mouse model (*n* = 3). b) Liver tissue section post 4 h administration of Cy5.5‐loaded PBP‐NPs. Section thickness = 6 µm. Scale bar = 400 µm. c) Flow cytometry analysis of liver cells from high‐fat/high‐fructose diet‐induced NASH mouse model. Free dye and Cy5.5‐loaded nanoparticles were intravenously injected, and livers were harvested after 24 h (*n* = 3). d) Relative mRNA expression levels of SREBP1c, DGAT2, and PHB in AML12 cell after incubating with FFAs at different concentrations for 48 h (*n* = 4). e) Prohibitin‐mediated cellular uptake of PBP‐NPs in fatty AML12 cells was analyzed by flow cytometry following 1 µg (red) and 3 µg (yellow) of antiprohibitin antibody (aPHB) preincubation for 2 h. Nonbinding peptide, NBP (CGGRGAGAC), was compared as a negative control (*n* = 4). f) Relative mRNA expression levels of HO‐1 in fatty AML12 cells. The mRNA levels were normalized to that of GAPDH mRNA by qPCR (*n* = 4). g) Relative mRNA expression levels of SIRT1, lipid metabolism marker (SREBP1c and FASN), and hepatic mitochondrial biogenesis markers (PGC1*α* and PPAR*α*) in fatty AML12 cells. The mRNA levels were normalized to that of GAPDH mRNA by qPCR (*n* = 4). h) Triglyceride levels in fatty AML12 cells after PBP‐NPs treatments were measured (*n* = 8). Data are presented as means ± SD. ns = not significant, *p < 0.033, **p < 0.01, ***p < 0.001, by one‐way ANOVA with Tukey's post hoc test were considered.

To study the biodistribution of PBP‐NPs in a NASH model, Cy5.5‐loaded PBP‐NPs were intravenously injected into the mouse fed a high‐fat/high‐fructose diet (HFHFD) for 28 weeks. Biodistribution kinetics with Cy5.5‐loaded PBP‐PLGA NPs in the NASH model was different from the high‐fat diet‐induced type 2 diabetic mouse model (Figure 4a and **Figure** **6**a). High localization of PBP‐NPs was observed in vWAT as well as the T2DM model until 24 h (Figure 6a and Figure S10, Supporting Information). However, PBP‐NPs were also highly distributed in the fatty liver of the NASH model than what was observed in the liver of the T2DM model (Figure 6a and Figure S10, Supporting Information). The high signal was also obtained in the cryosection of fatty liver tissue (Figure 6b and Figure S11, Supporting Information). Also, liver tissues isolated from a high‐fat diet‐induced type 2 diabetic mouse model after injecting free dye and various Cy5.5‐loaded nanoparticles were analyzed using flow cytometry. Hepatocytes and hepatic nonparenchymal cells from collagenase‐incubated tissues were separated by serial centrifugation. *α*‐1‐antitrypsin (A1AT), primarily synthesized by hepatocytes, was used as selection markers for hepatocytes and cd11b for hepatic macrophages. As a result, PBP‐NPs treated groups showed higher populations with Cy5.5‐positive signals than PLGA NPs and NBP‐NPs treated groups (Figure 4c and Figure S12, Supporting Information). Taken together, PBP‐NPs were able to target hepatic macrophages and hepatocytes actively, and we hypothesized fatty liver of the NASH model has higher expression of PHB than other obesity‐induced metabolic syndrome models.

To further investigate the different distribution of PBP‐NPs in the NASH model, protein expressions of the liver from different metabolic syndrome models were evaluated. Total proteins were isolated from the livers of mice with different diets and feeding times to make different degrades of fatty liver. Interestingly, levels of PHB expression were higher when additionally fed with 40% fructose and a longer intake of high calories diet (Figure S13, Supporting Information). In addition, AML 12, a murine normal hepatocytes cell line, incubated with FFAs showed higher gene expression of lipid metabolism markers (SREBP1c), triglycerides synthesizing enzyme (diacylglycerol O‐acyltransferase 2, DGAT2), and PHB as concentrations of FFAs increasing (Figure 6d). Furthermore, the PBP‐NPs targetability of hepatocytes via PHB binding was analyzed using FFAs‐incubated AML12 cells. Cy5.5‐loaded PBP‐NPs were found to selectively target hepatocytes via prohibitin binding and pretreatment with aPHB reduced uptake of PBP‐NPs into ATM in a dose‐dependent manner (Figure 6e). Taken together, consequent uptakes of free fatty acids in liver induced overexpression of PHB and PBP‐NPs could target vWAT and liver simultaneously in the NASH model.

As PBP‐NPs were demonstrated to specifically deliver drugs into hepatocytes, therapeutic effects of PBP‐NPs were conducted using FFAs‐incubated AML12 cells. Higher expression of HO‐1 was obtained in the HO‐1 inducer‐loaded PBP‐NPs groups compared with free drugs, PLGA NPs containing drugs, and PBP‐NPs not containing drugs (Figure 6f). Furthermore, HO‐1 inducer‐loaded PBP‐NPs groups inhibit lipid storage‐related genes (SREBP1c and FASN) expression and elevated mitochondrial biogenesis markers (PGC1*α* and PPAR*α*) than other groups (Figure 6g). Finally, lowered lipid storage and boosted mitochondrial activities resulted in hepatic triglycerides reduction without toxicity (Figure 6h and Figure S14, Supporting Information). In conclusion, PBP‐NPs have potential to alter the lipid metabolism of fatty hepatocytes and make synergistic effects by simultaneously reducing circulating FFAs and cytokines secreted by adipose tissue.

PBP‐NPs containing HO‐1 inducers were injected intravenously once a week for 4 weeks and analyzed to demonstrate synergistic effects of fatty liver/adipose tissue dual‐targeting PBP‐NPs in the NASH model. HO‐1‐induced groups significantly decreased body weight and fat mass after injection without the problem of calorie intake (Figures S15–S17, Supporting Information). Furthermore, PBP‐NPs stimulated HO‐1 expression, brown adipogenesis, and mitochondrial activity in the vWAT of the NASH model (Figure S18, Supporting Information). Also, differentiated brown adipocytes increased the activity of UCP1 to increase energy expenditure as shown in immunohistochemistry images (Figure S19, Supporting Information). Consequently, circulating FFAs, triglycerides, and cholesterol were significantly decreased in blood samples (Figure S20, Supporting Information). Inflammatory cytokines secreted by visceral adipose tissue were also reduced in HO‐1‐induced adipose tissues (Figures S21 and S22, Supporting Information). Taken together, PBP‐NPs were proven to be able to modulate adipose tissue environments of the NASH model like the obese diabetic model.

**Figure 7:**
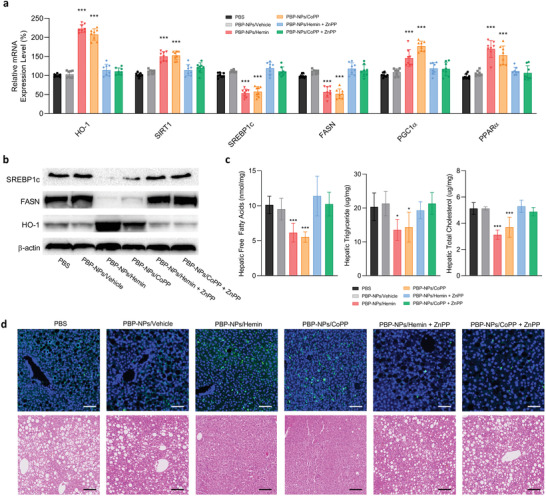
Normalization of lipid metabolism by PBP‐NPs in a high‐fat/high‐fructose diet‐induced NASH mouse model. a) Relative mRNA expression levels of HO‐1, SIRT1, lipid metabolism marker (SREBP1c and FASN), and hepatic mitochondrial biogenesis markers (PGC1*α* and PPAR*α*) in the fatty liver. The mRNA levels were normalized to that of GAPDH mRNA by qPCR (*n* = 8). b) Western blot image of HO‐1, SREBP1c, and FASN of liver lysates. c) Hepatic free fatty acids, triglyceride, and total cholesterol levels (*n* = 5). d) Immunofluorescence staining of HO‐1 (green) in the liver embedded in paraffin (top row) and representative image of H&E staining (bottom row). Section thickness = 6 µm. Scale bar = 200 µm. Data are presented as means ± SD. ns = not significant, *p < 0.033, **p < 0.01, ***p < 0.001, by one‐way ANOVA with Tukey's post hoc test were considered.

Additionally, HO‐1 expression was increased in the livers of the high‐fat high‐fructose diet‐induced NASH mouse model injected with PBP‐NPs/Hemin or PBP‐NPs/CoPP (**Figure** **7**a). The expression level of HO‐1 in NASH models’ liver was extremely higher than in T2DM models’ liver, indicating direct delivery of PBP‐NPs via PHB in fatty liver of NASH was significantly efficient (Figure 7a and Figure S22, Supporting Information). HO‐1 gene expression level in liver from T2DM mice was increased, but not enough to induce changes in downstream genes such as SIRT1, SREBP1c, and FASN (Figure S22, Supporting Information). On the other side, mRNA expressions of lipid metabolism markers (SREBP1c and FASN) that facilitate the accumulation of lipids were highly reduced than the T2DM model (Figure 7a and Figure S22, Supporting Information). Furthermore, protein levels of SREBP1c and FASN analyzed by western blot were also significantly decreased (Figure 7b). In addition, hepatic mitochondrial biogenesis markers (PGC1*α* and PPAR*α*) were elevated to boost fatty acid beta‐oxidation, which resulted in decreased hepatic free fatty acids, triglyceride, and total cholesterol levels (Figure 7a,c). Finally, immunofluorescence and H&E staining showed that lowered serum FFAs levels exhausted by brown adipose tissue and increased HO‐1 activity in the liver synergistically alleviated hepatic steatosis due to the reduction of hepatic ballooning and hepatic lipid accumulation in PBP‐NPs injected mice (Figure 7d).

Hepatic macrophages are composed of liver resident Kupffer cells and monocyte‐derived macrophages, which rapidly infiltrate the liver during injury.^[^ ^50^ ^]^ Under different disease conditions, the tissue microenvironmental cues of the liver critically influence the phenotypes and functions of hepatic macrophages.^[^ ^51^ ^]^ Furthermore, hepatic macrophages interact with multiple cell types in the liver, such as hepatocytes, neutrophils, endothelial cells, and platelets.^[^ ^50^ ^]^ These crosstalk interactions play important roles in regulating the extents of liver injury, repair, and ultimately liver disease progression. Hepatic macrophages have also been reported to modulate hepatic stellate cell (HSC) functions through TGF‐*β* induction resulting in liver fibrosis.^[^ ^52^, ^53^ ^]^

**Figure 8:**
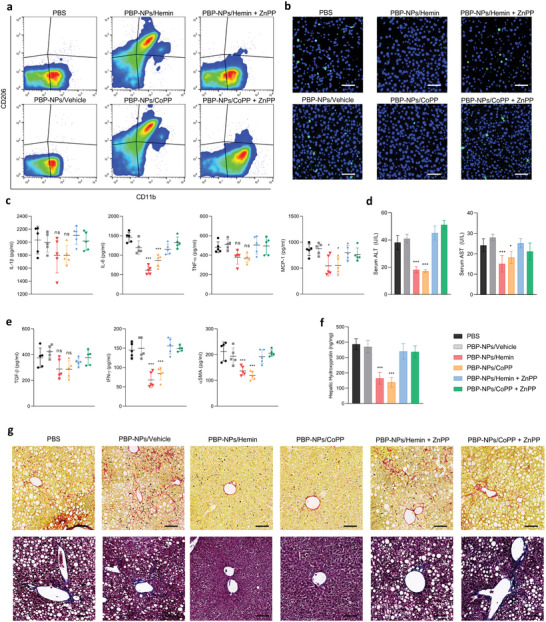
Anti‐inflammation and antifibrosis effects via M2 Kupffer cell differentiation of PBP‐NPs in the high‐fat/high‐fructose diet‐induced NASH mouse model. a) Phenotypes of macrophages isolated from the liver were analyzed by flow cytometry (*n* = 3). b) Hepatocytes in the apoptosis stage were measured by TUNEL staining of sectioned liver tissue. Scale bar = 100 µm. c) Inflammatory cytokines in the liver were analyzed by ELISA. d) ALT and AST were analyzed by ELISA (*n* = 5). e) Hepatic stellate cell‐activating cytokines were analyzed by ELISA for measuring fibrogenic phenomena. f) Hydroxyproline formed upon hydrolysis of connective‐tissue proteins was measured in the liver tissue (*n* = 5). g) Representative histological images of liver tissue sections stained with Sirius Red and Masson's Trichrome for collagen (red and blue) observation. Section thickness = 6 µm. Scale bar = 200 µm. Data are presented as means ± SD. ns = not significant, *p < 0.033, **p < 0.01, ***p < 0.001, by one‐way ANOVA with Tukey's post hoc test were considered.

We isolated monocyte populations from the NASH liver after treatments and analyzed them with flow cytometry. The CD206+ monocyte population was significantly increased in PBP‐NPs injected groups (**Figure** **8**a). Furthermore, proinflammatory cytokines such as IL‐1beta, IL‐6, TNF‐alpha, and MCP‐1 were decreased in HO‐1 induced liver, and PBP‐NPs also reduced hepatic apoptosis measured by immunofluorescence terminal deoxynucleotidyl transferase dUTP nick end labeling (TUNEL) staining (Figure 8b,c). Serum aspartate aminotransferase (AST) and alanine aminotransferase (ALT), indicators of liver failure, were also decreased after treatments (Figure 8d). As M1 Kupffer cells modulate fibrogenic responses of HSCs, activation markers of HSCs (TGF‐beta, IFN‐gamma, and a‐SMA) were measured by enzyme‐linked immunosorbent assay (ELISA). Activation markers of HSCs and hydroxyproline, a main component of collagen, were decreased in PBP‐NPs treated groups (Figure 8e,f). Finally, high levels of collagen inducing hepatic fibrosis around the microvessel in the liver were reduced (Figure 8g). Taken together, PBP‐NPs could directly target injured fatty liver to inhibit lipid accumulation in hepatocytes, decrease hepatic inflammation, and deactivate hepatic fibrosis induced by M1 Kupffer cells. These effects in the fatty liver could be more efficient because of the remodeling of adipose tissue simultaneously by inhibiting the secretion of lipids and cytokines (Figures S20–S22, Supporting Information).

## 3 Discussion

Therapeutic agents administered systemically often result in unwanted side effects and lowered efficacy owing to their accumulation in off‐target sites. Peptides with good stability, low cost, ease of manipulation, and large‐scale production could be unique gadgets in guiding polymeric nanomedicines to accomplish efficient and safe drug delivery.^[^ ^54^ ^]^ Peptide‐modified nanoparticles have demonstrated prolonged circulation, enhanced cellular uptake and targetability, and/or effective cytosolic drug release to achieve high efficacy both in vitro and in vivo.^[^ ^55^, ^56^, ^57^, ^58^ ^]^ We developed HO‐1 inducer‐loaded PLGA nanoparticles with PBP conjugation. PBP is a 9‐mer peptide (CKGGRAKDC) with cysteine in both ends and has a selective affinity to prohibitin that is frequently expressed on the membrane of target cells. PBP was conjugated with PEG through the reaction between maleimide and cysteine, and PEGylated peptide was coupled to PLGA nanoparticles via EDC/sulfo‐NHS reaction (Figure 1).

HO‐1 has been reported to provide the anti‐inflammatory and antiapoptotic effects in various obesity‐induced metabolic syndromes.^[^ ^15^, ^21^, ^24^, ^26^, ^27^, ^28^ ^]^ The expression level of HO‑1 is typically induced by ROS and strongly controlled by a redox‑sensitive transcription factor such as Nrf2.^[^ ^59^ ^]^ Thus, introducing HO‐1 by delivering mRNA, DNA, or protein without any coordinated transcription factor is not sufficient to obtain long‐last protective effects of HO‐1 and often causes a relapse of inflammation.^[^ ^60^ ^]^ However, small molecule inducers like hemin and CoPP separate Nrf2 from Kelch‑like ECH‑associated protein 1 (KEAP1) and transfer it into the nucleus to stimulate HO‐1 expression.^[^ ^38^ ^]^ For this mechanism, HO‐1 upregulation by hemin and CoPP is advantageous and long‐last enough to modulate environments of obesity and obesity‐induced metabolic syndrome. We loaded hemin and CoPP as HO‐1 inducers in PBP‐NPs and delivered them to prohibitin‐expressing cells and tissues. PBP‐NPs were able to successfully target white adipocytes and inflammatory macrophages in fatty adipose tissues (Figures 2, –5). PBP‐NPs activated thermogenic pathways to differentiate lipid storing white adipocytes into brown adipocytes to generate energy by exhausting lipids (Figures 2 and 4). Simultaneously, PBP‐NPs induced cytokine‐producing proinflammatory M1 macrophages to switch their phenotype into anti‐inflammatory M2 macrophages accelerating brown adipogenesis and tissue repair (Figures 3 and 5). Taken together, PBP‐NPs with HO‐1 inducers were proven to treat obesity and high‐fat diet‐induced type 2 diabetes through clearing free fatty acids and cytokines produced by fatty adipose tissue environments.

**Figure 9:**
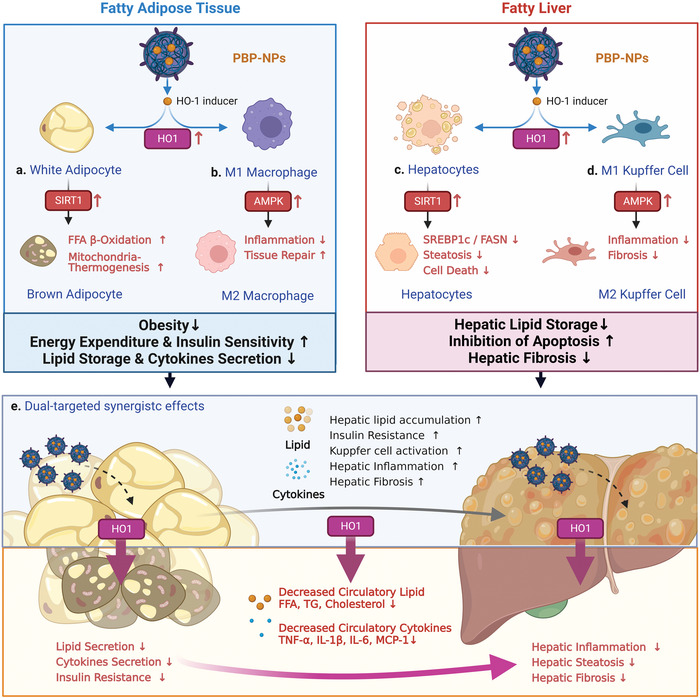
Summarizing schematic illustration of fatty liver/adipose tissue dual‐targeting PBP‐NPs containing HO‐1 inducers for the treatment of obesity, obesity‐induced type 2 diabetes, and nonalcoholic steatohepatitis. a) PBP‐NPs target adipocytes and induce brown adipogenesis via the SIRT1 pathway. Activated brown adipocytes accelerate mitochondrial biogenesis and turned fatty acids into energy. b) PBP‐NPs also switch inflammatory cytokines secreting M1 macrophages into anti‐inflammatory M2 via AMPK signaling. c) PBP‐NPs target the fatty liver to inhibit fatty acids storage by FASN and SREBP1c downregulation and ameliorate hepatic steatosis. d) HO‐1 expression also switches M1 Kupffer cells into M2 Kupffer cells to stop secreting inflammatory cytokines and activating hepatic stellate cells. e) Circulatory lipids and cytokines from obese adipose tissue are significantly reduced by PBP‐NPs and synergistically reduce hepatic steatosis, inflammation, and fibrosis. The figure was created with BioRender.com.

Furthermore, we confirmed overexpression of prohibitin in the fatty liver and synergistic effects of PBP‐NPs by targeting the fatty liver and adipose tissues simultaneously. PBP‐NPs in the NASH model not only cleared circulating free fatty acids and cytokines from adipose tissue, but also directly modified fatty hepatic environments (Figures 6 and 7). Overexpressed HO‐1 in the fatty liver inhibited lipid storage by downregulating FASN and SREBP1c in hepatocytes (Figures 6 and 7). Recovery of hepatic ballooning (enlarged lipid droplets) was accelerated by a combination of direct effects in hepatocytes and indirect effects decreasing serum free fatty acids secreted from fatty adipose tissues. Also, PBP‐NPs differentiated hepatic macrophages into M2 types by directly overexpressing HO‐1 in the liver and indirectly decreasing circulating free fatty acids and cytokines secreted from fatty adipose tissues (Figure 8). These synergistic effects ameliorated hepatic steatosis, inflammation, and fibrosis in the high‐fat high‐fructose diet‐induced NASH mouse model and overall conclusions are summarized in **Figure** **9**.

Due to the complexity of the pathophysiology of obesity‐induced metabolic syndromes, remedies modulating a single molecular target or symptom may not be successful. For example, farnesoid X receptor (FXR) agonists were able to modulate hepatic metabolism, and reduce hepatic glucogenesis and lipogenesis in NASH, but showed elevated FFAs and LDL cholesterol levels in serum, which could regenerate hepatic steatosis.^[^ ^61^ ^]^ In the present study, we demonstrated that dual‐targeted induction of heme oxygenase‐1 in fatty adipocytes and hepatocytes by PBP‐NPs synergistically ameliorated obesity, insulin resistance, inflammation, and steatohepatitis. The PBP‐NPs containing HO‐1 inducers have diverse therapeutic advantages that could be achieved simultaneously: 1) avoiding off target effects of HO‐1, 2) inducing brown adipogenesis to exhaust circulating lipids, 3) lowering systemic inflammation caused by obese ATMs, 4) decreasing hepatic accumulation of lipids and cytokines, 5) normalizing hepatic lipid metabolism and protecting from hepatic apoptosis, 6) inhibiting activation of hepatic macrophages to initiate hepatic fibrosis. Overall, dual targeting and multifunctional PBP‐NPs containing HO‐1 inducers were proven to be efficient therapeutics for downregulating disease‐causing molecular targets in injured organs. Material wise, PBP‐NPs have favorable properties for clinical application. Hemin (PANHEMATIN) is an FDA‐approved drug for the treatment of repeated attacks of acute intermittent porphyria and PLGA has been widely used in many FDA‐approved drug products. In this respect, PBP‐NPs containing HO‐1 inducers have a strong potential as safe and efficient nanotherapeutics for curing and preventing obesity, obesity‐induced metabolic syndromes, and NASH in future clinical studies.

## 4 Experimental Section

### Materials

Hemin and cobalt protoporphyrin ix were obtained from Persian Sigma‐Aldrich (Carlsbad, USA). Resomer PLGA 502 H with molecular weight between 7000 and 17 000 was obtained from Sigma‐Aldrich (Carlsbad, USA). Maleimide‐PEG‐amine (MW 2000) was purchased from Jenkem (Tianjin, China). Sulfo‐NHS and EDC were purchased from Sigma‐Aldrich (Carlsbad, USA). The PBP peptide (CKGGRAKDC) was synthesized by Peptron (Daejeon, Korea).

### Preparation of PBP‐NPs

Hemin or cobalt protoporphyrin ix (CoPP) (3 mg) and Resomer PLGA 502 H (125 mg) were dissolved in 2.5 mL of DMSO. The solution was agitated for 1 h and dripped into stirring 20 mL of 4% PVA solution slowly. For dispersion of small vesicles, the solution was sonicated for 20 s at 20% amplitude and stirred for 6 h at room temperature (RT) in the fume hood until all organic solvents evaporate. Finally, PLGA NPs were washed three times with distilled water by centrifugation and freeze‐dried. PBP peptides (10 mg) were incubated with acetic anhydride for 1 h at RT with an equal molar ratio and undergo dialysis with phosphate‐buffered saline (PBS) overnight. Then maleimide‐PEG (2000)‐amine was added into N‐terminal blocked peptides to create a thioether bond between the thiol group of cysteine and maleimide. After 24 h at 4 °C, PD‐10 desalting column was used for changing the PBS buffer into 0.1 m MES buffer of modified peptides. Finally, PLGA NPs dissolved in 0.1 m MES buffer incubated with 0.3 × 10^−3^ m EDC for 30 min and 0.15 × 10^−3^ m sulfo‐NHS for 2 h to conjugate with PBP‐PEG‐NH2. The equal molar ratio of PBP‐PEG‐NH2 was added to activated PLGA NPs and incubated for 3 h at RT. Finally, HO‐1 inducer‐loaded PBP‐NPs were centrifuged for 15 min at 18 000 RPM and washed two times with distilled water to be used.

### Drug Loading, Encapsulation Efficiency, and Cumulative Drug Release

PBP‐NPs containing hemin or CoPP (1 mg) were dissolved in 1 mL of ACN/DCM solution (v/v = 2:1). The concentrations of hemin and CoPP were detected by measuring absorbance at *λ*  =  630 and 580 nm, relatively (TECAN, Korea). Finally, the drug loading and encapsulation efficiency were calculated using the equations below. To study drug release kinetics, 10 mg of PBP‐NPs were dispersed in 5 mL PBS (pH 7.4) in a dialysis bag and dialyzed in 50 mL PBS at 37 °C. At each time point, the whole media were collected and replaced with fresh PBS, and the concentrations of hemin and CoPP were detected by measuring absorbance at *λ*  =  630 and 580 nm, relatively (TECAN, Korea)

(1) $$\begin{array}{c} \mathrm{Drug}\;\mathrm{loading}=\frac{\mathrm{weight}\;\mathrm{of}\;\mathrm{HO}-1\;\mathrm{inducer}\;\mathrm{in}\;\mathrm{PBP}-\mathrm{NPs}\;(\mathrm{mg})}{\mathrm{weight}\;\mathrm{of}\;\mathrm{PBP}-\mathrm{NPs}\;(\mathrm{mg})}\times 100\% \end{array}$$

(2) $$\begin{array}{ccc} & & \mathrm{Encapsulation}\;\mathrm{efficiency}\\ & & \;=\frac{\mathrm{weight}\;\mathrm{of}\;\mathrm{HO}-1\;\mathrm{inducer}\;\mathrm{in}\;\mathrm{PBP}-\mathrm{NPs}\;(\mathrm{mg})}{\mathrm{total}\;\mathrm{added}\;\mathrm{weight}\;\mathrm{of}\;\mathrm{HO}-1\;\mathrm{inducer}\;\mathrm{for}\;\mathrm{PBP}-\mathrm{NPs}\;\mathrm{preparation}\;(\mathrm{mg})}\\ & & \;\;\;\;\times \;100\% \end{array}$$

### Cell Line

3T3‐L1 cells and AML12 cells were purchased from ATCC (Virginia, USA). High glucose Dulbecco's modified Eagle medium purchased from WelGENE (Seoul, Korea) with 10% fetal bovine serum and 1% penicillin‐streptomycin (100 U mL^−1^) was used for cell culture. 3T3‐L1 was incubated at 37 °C in 5% CO_2_ of the atmosphere and passed every other day. For adipocyte differentiation, cells were treated with a differentiation medium containing a complete medium, 10 µg mL^−1^ insulin, 1 × 10^−6^ m dexamethasone, and 0.5 × 10^−3^ m IBMX for 72 h. The differentiation medium was replaced with a complete medium containing 10 µg mL^−1^ insulin and the complete medium was changed every 2 days. After 10 days, mature adipocytes were analyzed. For fatty acid incubation for hepatocytes, AML12 were treated with a mixture of oleic and palmitic acids with a volume ratio of 1:2 for 48 h.

### High‐Fat Diet‐Induced Type 2 Diabetic Mouse Model

6 weeks old male C57BL/6J mice were obtained from Orient Bio (Seongnam, Korea). All experimental procedures were approved by the Institutional Animal Care and Use Committee at Hanyang University (2020‐0222A). All mice were housed under specific pathogen‐free conditions and fed normal chow (Orient Bio) for the first 2 weeks and from the 3rd week, the diet was mixed with 60% Kcal high‐fat diets (high‐fat diet, HFD, Central Lab Animal, Inc.). The proportion of the HFD in the total rodent diet was gradually increased for the 6th week and the mice were fed with HFD for 8 more weeks. Mice became obese and insulin‐resistant after the 15th week, with an approximate bodyweight of 45–55 g and fasted glucose level >200 mg dL^−1^.

### High‐Fat High‐Fructose Diet‐Induced NASH Mouse Model

To induce NASH in the C57BL/6J, 6 weeks old male C57BL/6J mice from Orient Bio were fed with normal chow (Orient Bio) for the first 2 weeks and after then, the diet was mixed with 60% Kcal high‐fat diets (High‐fat diet, HFD, Central Lab Animal, Inc.). The proportion of HFD in the total rodent diet was gradually increased for the 6th week. Simultaneously, 10% D‐fructose in autoclaved tap water was given to mice from the 3rd week and gradually increased the concertation of D‐fructose to 25% for the 6th week. The mice were fed with a HFHFD for 28 weeks. The NASH was induced after the 30th week, with an approximate bodyweight of 55–60 g and fasted glucose level >250 mg dL^−1^.

### In Vivo Biodistribution

The Cy5.5‐loaded PBP‐NPs were injected intravenously into mice. Mice were sacrificed at 0, 1, 4, and 24 h of injection, and the liver, spleen, kidneys, lungs, heart, visceral adipose tissues, and subcutaneous adipose tissues were harvested. Ex vivo Cy5.5 fluorescence in each organ was detected using the VISQUE In Vivo Smart Fluorescence device of the Korea Basic Science Institute (Chuncheon, Korea) by measuring the mean fluorescence intensity per tissue area.

### Animal Study

After the preparation of a high‐fat diet‐induced type 2 diabetic mouse model and a high‐fat high‐fructose diet‐induced NASH mouse model, 1 mg kg^−1^ of HO‐1 inducer‐loaded PBP‐NPs were intravenously injected through the tail vein once a week. The HO‐1 inhibitor (ZnPP) (0.25 mg kg^−1^) was intraperitoneally injected to compete with PBP‐NPs effects. After 4 weeks of treatments, mice were observed 3 weeks more for further therapeutic effects and sacrificed at the 8th week for ex vivo analysis.

### Insulin Tolerance Test

The initial blood glucose level was measured at 6 h post‐fasting using the Accu‐Chek Active model GC kit (RocheDiagnostics GmbH, USA). Insulin (0.75 Unit kg^−1^) was injected intraperitoneally. Blood samples were collected at 0, 30, 60, 90, and 120 min post‐injection for the insulin tolerance test.

### Ex Vivo Analysis of mRNA

The samples from visceral white adipose tissue and liver were mechanically chopped and incubated with a collagenase solution for 1 h at 37 °C. The homogenates were centrifuged at 400 × *g* for 10 min to isolate cells and total RNA was extracted. After preparing equal amounts of cDNA from the extracted RNA, the relative gene expression levels were quantified by RT‐PCR (Applied Biosystems 7500, USA). Each sample was compared to the GAPDH control and calculated using the ΔΔCt method.

### Immunofluorescence Assay of Adipose Tissue

Adipose tissues were fixed in 4% paraformaldehyde, prepared as paraffin blocks, and sectioned at 8 µm. Paraffin blocks were deparaffinized and rehydrated in alcohol. Antibody against target protein was incubated overnight. After washing the primary antibody, FITC‐conjugated anti‐rabbit antibody (Abcam, USA) was incubated for 2 h. Sealing‐stained slides were prepared by mounting a coverslip with a Dako Fluorescence Mounting Medium (DAKO, Denmark) and scanned with AxioScan.Z1 (Zeiss, Germany).

### Ex Vivo Analysis of Cytokines and Lipids

At the end of the study, blood samples were obtained via retro‐orbital bleeding and tissues were surgically harvested. Total cholesterol, triglyceride, and FFAs concentrations in plasma and tissue were determined enzymatically with kits (Abcam, USA). Total cytokine concentrations in plasma and tissue were determined by ELISA kits (InvivoGen, USA).

### Ex Vivo Macrophage Population Analysis

Visceral white adipose tissue (vWAT) was harvested for ex vivo analysis. Adipose tissue was incubated in presence of collagenase at 37 °C for 45 min to isolate the stromal vascular fraction (SVF). After centrifugation, total SVF cells were stained with anti‐F4/80 antibody, anti‐CD11b antibody, and anti‐CD206 antibody, and analyzed by using flow cytometry. The relative mean fluorescence intensity value for each macrophage was measured using flow cytometry.

### Histological Analysis and Immunohistochemical Staining of Liver

Liver tissues were fixed in 4% neutral buffered formalin and embedded in paraffin. Then, the paraffin sections were deparaffinized and rehydrated in gradual mixtures of ethanol/water. The paraffin sections were blocked with 5% of bovine serum albumin blocking solution for 1 h and incubated with anti‐HO‐1 antibody overnight. The next day, the sections were washed and mounted with a mounting solution including 4′,6‐diamidino‐2‐phenylindole. On the other side, the 6 µm of paraffin sections were stained with H&E, Masson's trichrome, and Sirius red for histological analysis.

### Statistical Analysis

All the data were represented as the mean ± SD. GraphPad Prism (version 8.02) for Windows (GraphPad Software) was used for statistical analysis. Comparisons among groups were performed by one‐way analysis of variance (ANOVA) followed by Tukey's multiple comparisons test. *p*‐Values less than 0.05 were considered significant (ns = not significant, *p < 0.033, **p < 0.01, ***p < 0.001). Detailed processing of data and sample size for each analysis are described in figure legends.

## Conflict of Interest

The authors declare no conflict of interest.

## Author Contributions

J.H. and Y.‐H.K. designed the experimental design and protocol. J.H. carried out the in vitro, in vivo, and ex vivo therapeutic assays. J.H. and Y.‐H.K. discussed the results and wrote the paper.

## Supporting information

Supporting Information

## Data Availability

The data that support the findings of this study are available from the corresponding author upon reasonable request.

## References

[1] M.‐A. Cornier , D. Dabelea , T. L. Hernandez , R. C. Lindstrom , A. J. Steig , N. R. Stob , R. E. Van Pelt , H. Wang , R. H. Eckel , Endocr. Rev. 2008, 29, 777. 18971485 10.1210/er.2008-0024 PMC5393149

[2] A. Guilherme , J. V. Virbasius , V. Puri , M. P. Czech , Nat. Rev. Mol. Cell Biol. 2008, 9, 367. 18401346 10.1038/nrm2391 PMC2886982

[3] F. Liu , J. He , H. Wang , D. Zhu , Y. Bi , Obes. Surg. 2020, 30, 5086. 33021706 10.1007/s11695-020-04983-6 PMC7719100

[4] F. Karpe , J. R. Dickmann , K. N. Frayn , Diabetes 2011, 60, 2441. 21948998 10.2337/db11-0425 PMC3178283

[5] Y. Kawano , D. E. Cohen , J. Gastroenterol. 2013, 48, 434. 23397118 10.1007/s00535-013-0758-5 PMC3633701

[6] H. W. Baynes , S. Mideksa , S. Ambachew , Adipocyte 2018, 7, 81. 29537934 10.1080/21623945.2018.1443662 PMC6152539

[7] G. Cavaliere , G. Trinchese , P. Bergamo , C. De Filippo , G. Mattace Raso , G. Gifuni , R. Putti , B. H. Moni , R. B. Canani , R. Meli , M. P. Mollica , PLoS One 2016, 11, e0149033. 26901315 10.1371/journal.pone.0149033 PMC4762694

[8] S. N. Randeria , G. J. A. Thomson , T. A. Nell , T. Roberts , E. Pretorius , Cardiovasc. Diabetol. 2019, 18, 72. 31164120 10.1186/s12933-019-0870-9 PMC6549308

[9] J. Shi , J. Fan , Q. Su , Z. Yang , Front. Endocrinol. 2019, 10, 703. 10.3389/fendo.2019.00703 PMC6833922 31736870

[10] J. Zhang , Y. Zhao , C. Xu , Y. Hong , H. Lu , J. Wu , Y. Chen , Sci. Rep. 2014, 4, 5832. 25060337 10.1038/srep05832 PMC5376058

[11] H. Tilg , T. E. Adolph , M. Dudek , P. Knolle , Nat. Metab. 2021, 3, 1596. 34931080 10.1038/s42255-021-00501-9

[12] J. C. Arroyave‐Ospina , Z. Wu , Y. Geng , H. Moshage , Antioxidants 2021, 10, 174. 33530432 10.3390/antiox10020174 PMC7911109

[13] P. Rada , Á. González‐Rodríguez , C. García‐Monzón , Á. M. Valverde , Cell Death Dis. 2020, 11, 802. 32978374 10.1038/s41419-020-03003-w PMC7519685

[14] D. H. Ipsen , J. Lykkesfeldt , P. Tveden‐Nyborg , Cell. Mol. Life Sci. 2018, 75, 3313. 29936596 10.1007/s00018-018-2860-6 PMC6105174

[15] J. A. Araujo , M. Zhang , F. Yin , Front. Pharmacol. 2012, 3, 119. 22833723 10.3389/fphar.2012.00119 PMC3400084

[16] N. K. Campbell , H. K. Fitzgerald , A. Dunne , Nat. Rev. Immunol. 2021, 21, 411. 33514947 10.1038/s41577-020-00491-x

[17] S. W. Ryter , A. M. K. Choi , Antioxid. Redox Signaling 2005, 7, 80. 10.1089/ars.2005.7.80 15650398

[18] S. P. Singh , I. Grant , A. Meissner , A. Kappas , N. G. Abraham , Horm. Mol. Biol. Clin. Invest. 2017, 31, 20170027. 10.1515/hmbci-2017-0027 28763300

[19] L. Liu , N. Puri , M. Raffaele , J. Schragenheim , S. P. Singh , J. A. Bradbury , L. Bellner , L. Vanella , D. C. Zeldin , J. Cao , N. G. Abraham , Prostaglandins Other Lipid Mediators 2018, 138, 1. 30041041 10.1016/j.prostaglandins.2018.07.004 PMC6314013

[20] A. Bartelt , J. Heeren , Nat. Rev. Endocrinol. 2014, 10, 24. 24146030 10.1038/nrendo.2013.204

[21] J. F. Ndisang , Mediators Inflammation 2010, 2010, 359732. 10.1155/2010/359732 PMC2872759 20508722

[22] N.‐J. Song , S.‐H. Chang , D. Y. Li , C. J. Villanueva , K. W. Park , Exp. Mol. Med. 2017, 49, e353. 28684864 10.1038/emm.2017.70 PMC5565954

[23] L. Vanella , K. Sodhi , D. H. Kim , N. Puri , M. Maheshwari , T. D. Hinds , L. Bellner , D. Goldstein , S. J. Peterson , J. I. Shapiro , N. G. Abraham , Stem Cell Res. Ther. 2013, 4, 28. 23497794 10.1186/scrt176 PMC3706794

[24] X. Liu , Y. Gao , M. Li , C. Geng , H. Xu , Y. Yang , Y. Guo , T. Jiao , F. Fang , Y. Chang , J. Hepatol. 2015, 63, 713. 26026874 10.1016/j.jhep.2015.05.018

[25] N. G. Abraham , J. M. Junge , G. S. Drummond , Trends Pharmacol. Sci. 2016, 37, 17. 26515032 10.1016/j.tips.2015.09.003 PMC5488647

[26] M. Li , D. H. Kim , P. L. Tsenovoy , S. J. Peterson , R. Rezzani , L. F. Rodella , W. S. Aronow , S. Ikehara , N. G. Abraham , Diabetes 2008, 57, 1526. 18375438 10.2337/db07-1764

[27] Y. Naito , T. Takagi , Y. Higashimura , Arch. Biochem. Biophys. 2014, 564, 83. 25241054 10.1016/j.abb.2014.09.005

[28] T. H. Tu , Y. Joe , H.‐S. Choi , H. T. Chung , R. Yu , Mediators Inflammation 2014, 2014, 290708. 10.1155/2014/290708 PMC4244973 25477711

[29] S. W. Ryter , Antioxidants 2022, 11, 555.35326205

[30] X. Chen , S.‐Y. Wei , J.‐S. Li , Q.‐F. Zhang , Y.‐X. Wang , S.‐L. Zhao , J. Yu , C. Wang , Y. Qin , Q.‐J. Wei , G.‐X. Lv , B. Li , PLoS One 2016, 11, e0147084. 26765329 10.1371/journal.pone.0147084 PMC4713170

[31] Y. Son , J. H. Lee , H.‐T. Chung , H.‐O. Pae , Oxid. Med. Cell. Longevity 2013, 2013, 639541. 10.1155/2013/639541 PMC3786516 24101950

[32] X. Zhang , M. Ding , P. Zhu , H. Huang , Q. Zhuang , J. Shen , Y. Cai , M. Zhao , Q. He , Oxid. Med. Cell. Longevity 2019, 2019, 3214196. 10.1155/2019/3214196 PMC6885770 31827672

[33] S. M. U. Ahmed , L. Luo , A. Namani , X. J. Wang , X. Tang , Biochim. Biophys. Acta, Mol. Basis Dis. 2017, 1863, 585. 27825853 10.1016/j.bbadis.2016.11.005

[34] J.‐F. Luo , X.‐Y. Shen , C. K. Lio , Y. Dai , C.‐S. Cheng , J.‐X. Liu , Y.‐D. Yao , Y. Yu , Y. Xie , P. Luo , X.‐S. Yao , Z.‐Q. Liu , H. Zhou , Front. Pharmacol. 2018, 9, 911. 30233360 10.3389/fphar.2018.00911 PMC6131578

[35] Z. Chen , H. Zhong , J. Wei , S. Lin , Z. Zong , F. Gong , X. Huang , J. Sun , P. Li , H. Lin , B. Wei , J. Chu , Arthritis Res. Ther. 2019, 21, 300. 31870428 10.1186/s13075-019-2085-6 PMC6929452

[36] N. F. Villeneuve , A. Lau , D. D. Zhang , Antioxid. Redox Signaling 2010, 13, 1699. 10.1089/ars.2010.3211 PMC2966484 20486766

[37] L. Baird , M. Yamamoto , Mol. Cell. Biol. 2020, 40, e00099. 32284348 10.1128/MCB.00099-20 PMC7296212

[38] K. Nurmi , I. Kareinen , J. Virkanen , K. Rajamäki , V.‐P. Kouri , K. Vaali , A.‐L. Levonen , N. Fyhrquist , S. Matikainen , P. T. Kovanen , K. K. Eklund , J. Innate Immun. 2017, 9, 65. 27655219 10.1159/000448894 PMC6738905

[39] R. Holland , J. C. Fishbein , Antioxid. Redox Signaling 2010, 13, 1749. 10.1089/ars.2010.3273 PMC2959180 20486763

[40] Y.‐W. Won , P. P. Adhikary , K. S. Lim , H. J. Kim , J. K. Kim , Y.‐H. Kim , Nat. Mater. 2014, 13, 1157. 25282508 10.1038/nmat4092

[41] D. Liu , Y. Lin , T. Kang , B. Huang , W. Xu , M. Garcia‐Barrio , M. Olatinwo , R. Matthews , Y. E. Chen , W. E. Thompson , PLoS One 2012, 7, e34315. 22479600 10.1371/journal.pone.0034315 PMC3316679

[42] S.‐B. Yong , Y. Song , Y.‐H. Kim , Biomaterials 2017, 148, 81. 28985514 10.1016/j.biomaterials.2017.09.023

[43] J. Y. Chung , Q. U. Ain , Y. Song , S.‐B. Yong , Y.‐H. Kim , Genome Res. 2019, 29, 1442. 31467027 10.1101/gr.246900.118 PMC6724665

[44] J. Y. Chung , J. Hong , H.‐J. Kim , Y. Song , S.‐B. Yong , J. Lee , Y.‐H. Kim , Biomaterials 2021, 279, 121209. 34700224 10.1016/j.biomaterials.2021.121209

[45] L. Xie , M. T. Ortega , S. Mora , S. K. Chapes , Clin. Vaccine Immunol. 2010, 17, 651. 20164250 10.1128/CVI.00494-09 PMC2849320

[46] R. Liu , B. S. Nikolajczyk , Front. Immunol. 2019, 10, 1587. 31379820 10.3389/fimmu.2019.01587 PMC6653202

[47] C. Peng , A. G. Stewart , O. L. Woodman , R. H. Ritchie , C. X. Qin , Front. Pharmacol. 2020, 11, 603926. 33343375 10.3389/fphar.2020.603926 PMC7745178

[48] C. Boutari , N. Perakakis , C. S. Mantzoros , Endocrinol. Metab. 2018, 33, 33. 10.3803/EnM.2018.33.1.33 PMC5874193 29589386

[49] S. L. Friedman , Gastroenterology 2008, 134, 1655. 18471545 10.1053/j.gastro.2008.03.003 PMC2888539

[50] Z. Shan , C. Ju , Front. Immunol. 2020, 11, 322. 32362892 10.3389/fimmu.2020.00322 PMC7180226

[51] Y. Wen , J. Lambrecht , C. Ju , F. Tacke , Cell Mol. Immunol. 2021, 18, 45. 33041338 10.1038/s41423-020-00558-8 PMC7852525

[52] B. Dewidar , C. Meyer , S. Dooley , A. N. Meindl‐Beinker , Cells 2019, 8, 1419. 31718044 10.3390/cells8111419 PMC6912224

[53] M. Matsuda , E. Seki , Semin. Liver Dis. 2020, 40, 307. 32242330 10.1055/s-0040-1708876 PMC7484001

[54] W.‐J. Jeong , J. Bu , L. J. Kubiatowicz , S. S. Chen , Y. Kim , S. Hong , Nano Convergence 2018, 5, 38. 30539365 10.1186/s40580-018-0170-1 PMC6289934

[55] J. Ha , M. Kim , Y. Lee , M. Lee , Nanoscale 2021, 13, 14745. 34474460 10.1039/d1nr03455c

[56] J. Lee , W. Son , J. Hong , Y. Song , C.‐S. Yang , Y.‐H. Kim , J. Controlled Release 2021, 336, 344. 10.1016/j.jconrel.2021.06.022 34147573

[57] K. Chung , I. Ullah , N. Kim , J. Lim , J. Shin , S. C. Lee , S. Jeon , S. H. Kim , P. Kumar , S.‐K. Lee , J. Drug Targeting 2020, 28, 617. 10.1080/1061186X.2019.1706095 31852284

[58] G. Kim , M. Kim , Y. Lee , J. W. Byun , D. W. Hwang , M. Lee , J. Controlled Release 2020, 317, 273. 10.1016/j.jconrel.2019.11.009 31730913

[59] A. Loboda , M. Damulewicz , E. Pyza , A. Jozkowicz , J. Dulak , Cell. Mol. Life Sci. 2016, 73, 3221. 27100828 10.1007/s00018-016-2223-0 PMC4967105

[60] C. Rempf , A. Ritter , S. Schrepfer , M. Freitag , T. Standl , A. Gottschalk , Artif. Cells, Blood Substitutes, Immobilization Biotechnol. 2008, 36, 34. 10.1080/10731190701857769 18293159

[61] V. Massafra , R. Pellicciari , A. Gioiello , S. W. C. Van Mil , Pharmacol. Ther. 2018, 191, 162. 29933033 10.1016/j.pharmthera.2018.06.009

