# Supplementary material for: Fatty Liver/Adipose Tissue Dual‐Targeting Nanoparticles with Heme Oxygenase‐1 Inducer for Amelioration of Obesity, Obesity‐Induced Type 2 Diabetes, and Steatohepatitis
Source: Adv Sci (Weinh). 2022 Oct 9;9(33):2203286. doi: 10.1002/advs.202203286 (PMC9685446; doi:10.1002/advs.202203286)
Supplement: Supplementary file 1 — Supporting Information [file ADVS-9-2203286-s001.pdf]

Fatty liver/adipose tissue dual-targeting nanoparticles with heme oxygenase-1 inducer for amelioration of obesity, obesity-induced type 2 diabetes, and steatohepatitis

Juhyeong Hong[1,2], Yong-Hee Kim[1,2*]

[1]*Department of Bioengineering, Institute for Bioengineering and Biopharmaceutical Research Hanyang University, 04763 Seoul, South Korea,* [2]*Education and Research Group for Biopharmaceutical Innovation Leader, Hanyang University, 04763 Seoul, South Korea*

*Correspondence addressed to

Address: 222, Wangsimni-ro, Seongdong-gu, ITBT Building 908, Seoul, 04763

Phone: +82-2-2220-2345

Fax: +822-2220-4342

E-mail: yongheekim@hanyang.ac.kr

Supporting Information

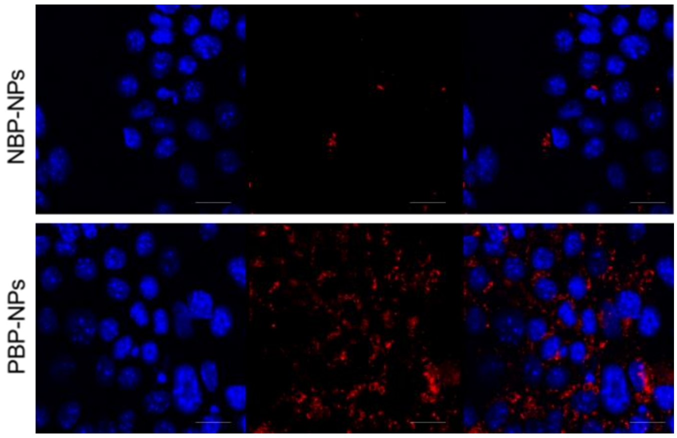

Figure S1. Selective uptake of PBP-NPs in 3T3-L1 cells. Comparison of cellular uptake between Cy5.5-loaded PBP-NPs and NBP-NPs following 24 h treatments. Nucleus (blue) and Cy5.5 (red) were visualized by CLSM. Scale bar = 20μm

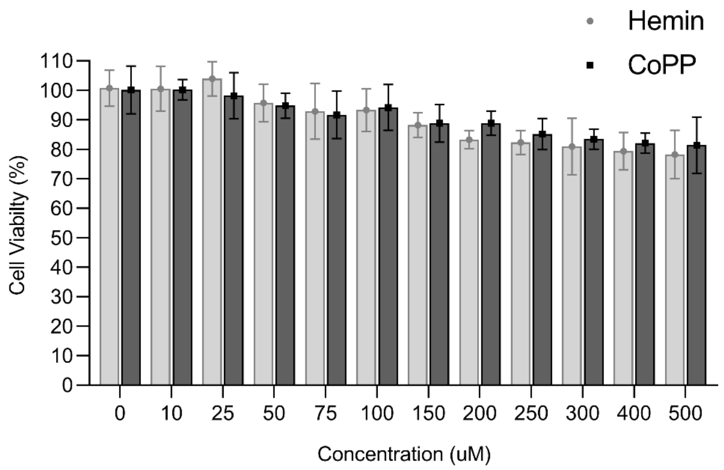

Figure S2. ***In vitro*** **cytotoxicity of PBP-NPs in 3T3-L1 cells.** Dose-dependent cell viability of PBP-NPs were evaluated by MTT assay in mature adipocytes. n=4. Data are presented as means ± SD.

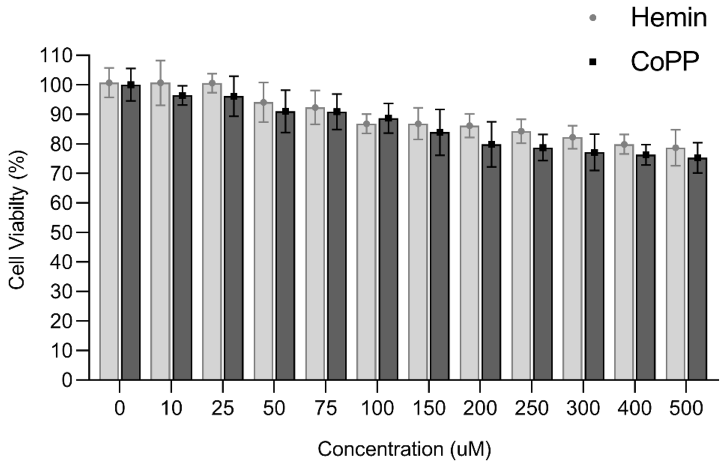

Figure S3. *In vitro* cytotoxicity of PBP-NPs in macrophages. Dose-dependent cell viability of PBP-NPs (Hemin, CoPP) were evaluated by MTT assay in adipose tissue-derived macrophages. n=4. Data are presented as means ± SD.

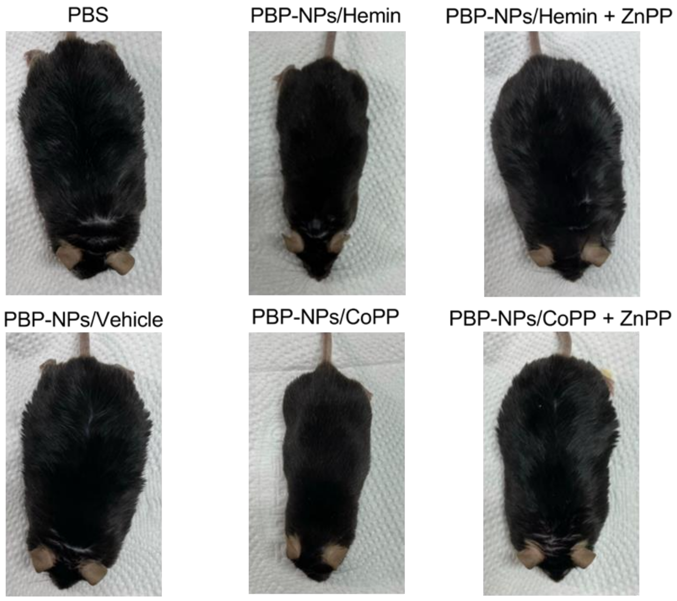

Figure S4. Representative images of the high-fat diet-induced type 2 diabetic mouse model after treatments. High-fat diet (HFD) fed mice were treated with PBP-NPs for 4 weeks and observed for additional 3 weeks. Representative images of the obesity-induced type 2 diabetes model were taken in the 8th week.

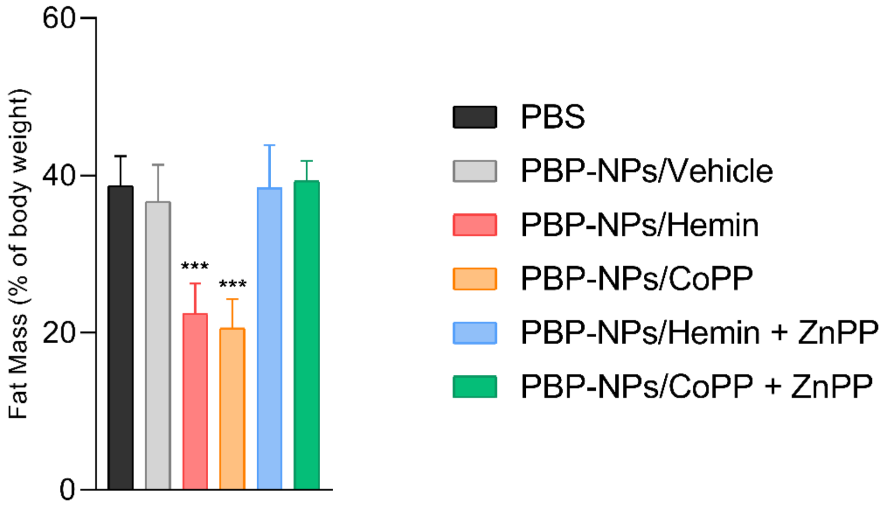

Figure S5. Measurement of total fat mass in a high-fat diet-induced type 2 diabetic mouse model after treatments. High-fat diet (HFD) fed mice were treated with PBP-NPs for 4 weeks and observed for additional 3 weeks. Amounts of total fat tissue were measured and normalized with body weights (n=5). Data are presented as means ± SD. ns = not significant, *p < 0.033, **p < 0.01, ***p < 0.001, by one-way ANOVA with Tukey's post hoc test were considered.

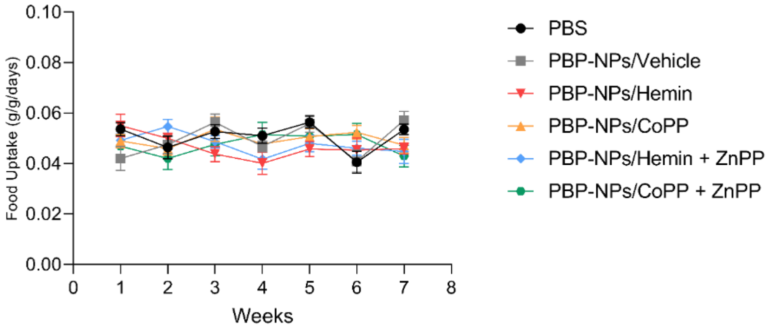

Figure S6. Patterns of weekly food uptake patterns in high-fat diet-induced type 2 diabetic mouse model after treatments. High-fat diet (HFD) fed mice were treated with PBP-NPs for 4 weeks and observed for additional 3 weeks. Amounts of HFD were measured every other day and normalized with the sum of the body weights of each group (n=3). Data are presented as means ± SD.

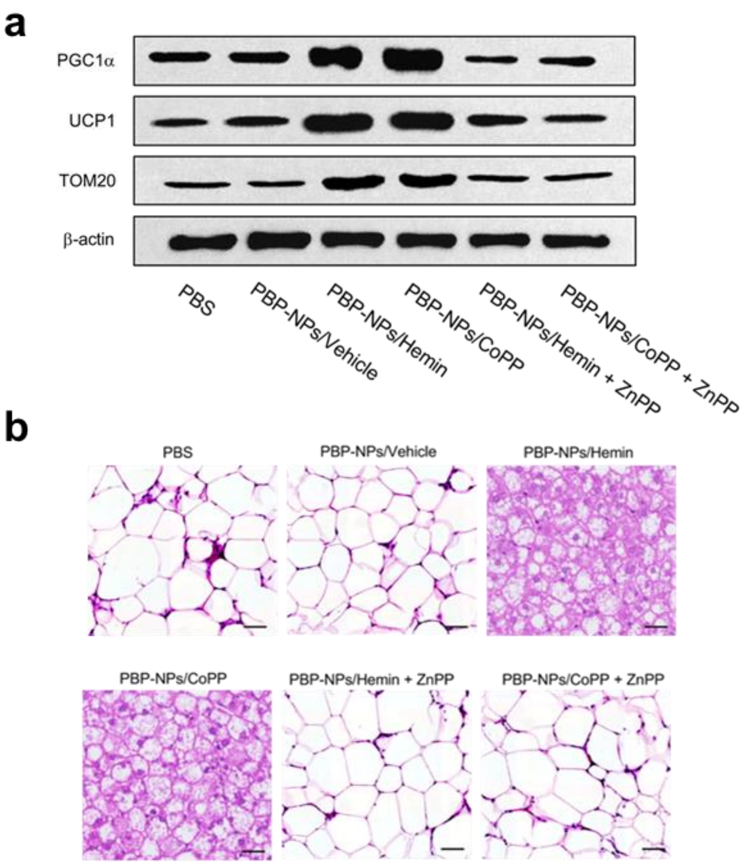

Figure S7. Protein levels analysis and morphological alternations of adipose tissue in a high-fat diet-induced type 2 diabetic mouse model after treatments. Following 4 weeks of treatments and additional 3 weeks of observation, visceral white adipose tissues were isolated. (a) Representative images of western blot analysis are shown. (b) Representative image of H&E staining in the adipose tissue embedded in paraffin. Section thickness = 6μm. Scale bar = 200μm.

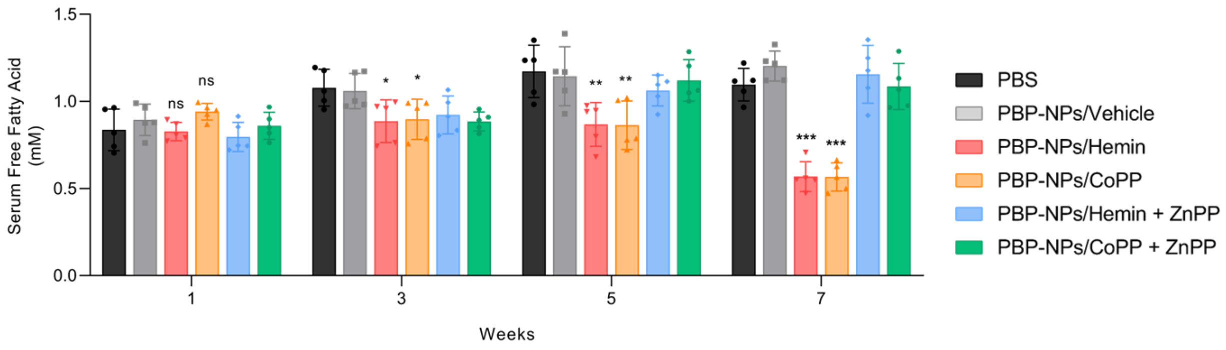

Figure S8. Serum levels of free fatty acids in a high-fat diet-induced type 2 diabetic mouse model after treatments. Following 4 weeks of treatments and additional 3 weeks of observation, blood samples were collected from the tail vein (n=5). The catheter was inserted into the vein and collected blood was transferred into a sterile empty tube. Coagulated blood samples were centrifuged 1000×g for 10 min at 4ºC to obtain serum. Data are presented as means ± SD. Data are presented as means ± SD. ns = not significant, *p < 0.033, **p < 0.01, ***p < 0.001, by one-way ANOVA with Tukey's post hoc test were considered.

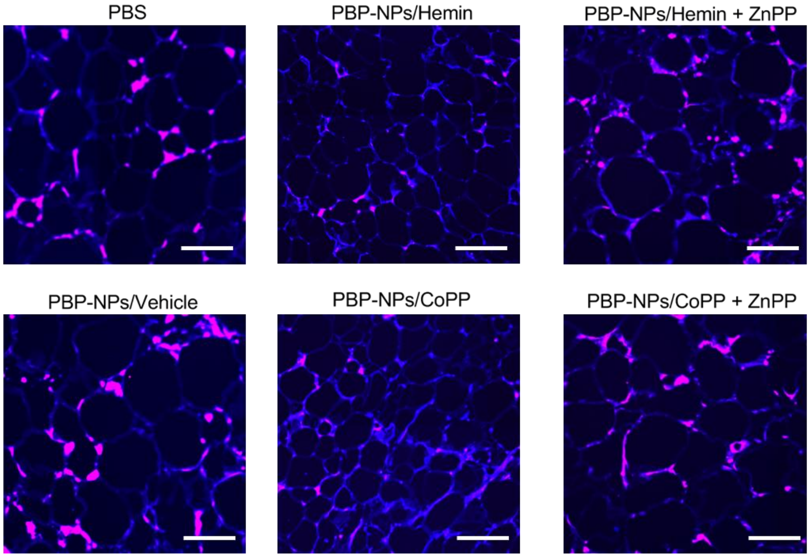

Figure S9. Reduction of crown-like structure (CLS) in visceral white adipose tissues of high-fat diet-induced type 2 diabetic mouse model after treatments. Crown-like structures (CLS) are histologic hallmarks of the infiltrated inflammatory macrophages in adipose tissue. HO-1 inducer-loaded PBP-NPs (1mg/kg) were intravenously injected through the tail vein once a week. The HO-1 inhibitor (ZnPP) (0.25mg/kg) were intraperitoneally injected to compete with PBP-NPs effects. Macrophages in visceral white adipose tissues were stained with an anti-F4/80 antibody (violet) and an anti-E-cadherin antibody (blue) was used for membrane staining. Scale bar = 200µm

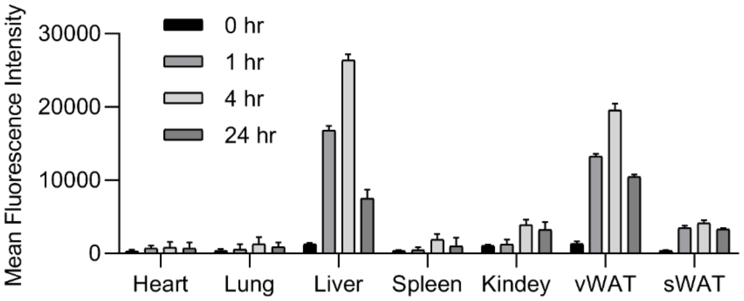

Figure S10. Biodistribution of intravenously injected Cy5.5-loaded PBP-NPs in the high-fat high-fructose diet-induced nonalcoholic steatohepatitis mouse model. Mean fluorescence intensity per tissue area of major organs (n=3). Data are presented as means ± SD.

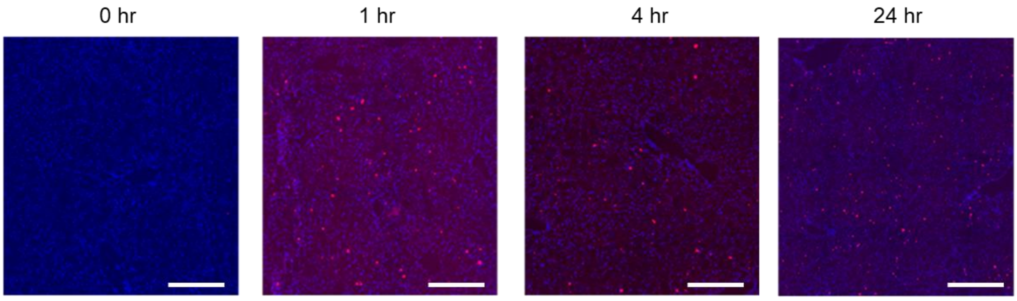

Figure S11. Effects of fatty liver-targeting by PBP-NPs in the high-fat high-fructose diet-induced nonalcoholic steatohepatitis mouse model. Liver tissues were obtained post 0, 1, 4, and 24 h administration of Cy5.5-loaded PBP-NPs. DAPI (blue) was used for nucleus staining. Section thickness = 6μm. Scale bar = 1000μm

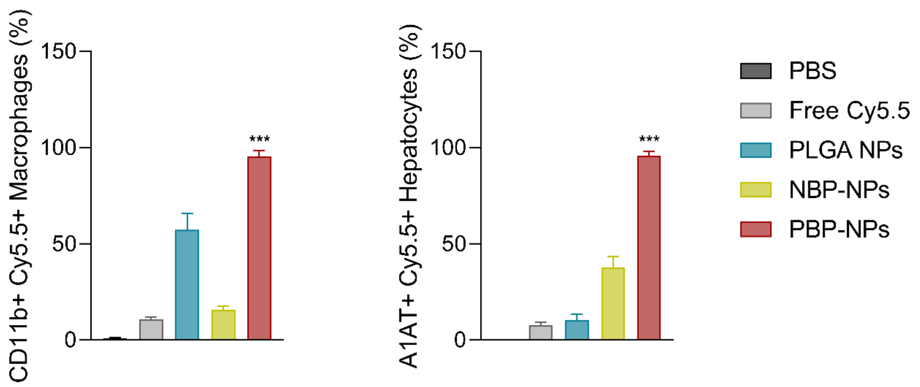

Figure S12. Macrophages and hepatocytes specific delivery of PBP-NPs in liver from the high-fat high-fructose diet-induced nonalcoholic steatohepatitis mouse model. Free dye and various Cy5.5-loaded nanoparticles were injected intravenously, and liver tissues were harvested after 24 h. CD11b+ macrophages and A1AT+ hepatocytes were analyzed by flow cytometry (n=3). Data are presented as means ± SD. ns = not significant, *p < 0.033, **p < 0.01, ***p < 0.001, by one-way ANOVA with Tukey's post hoc test were considered.

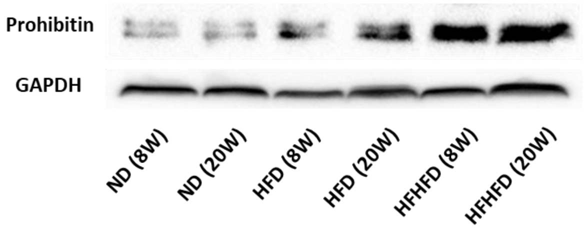

Figure S13. Expression levels of prohibitin in fatty liver tissues. Normal diet (ND), High-fat diet (HFD), and high-fructose high-fat diet (HFHFD) were fed to male C57BL/6 mice for 8 weeks and 20 weeks. Whole protein was extracted by RIPA lysis buffer from isolated livers and loaded on 12% acrylamide gel for western blot.

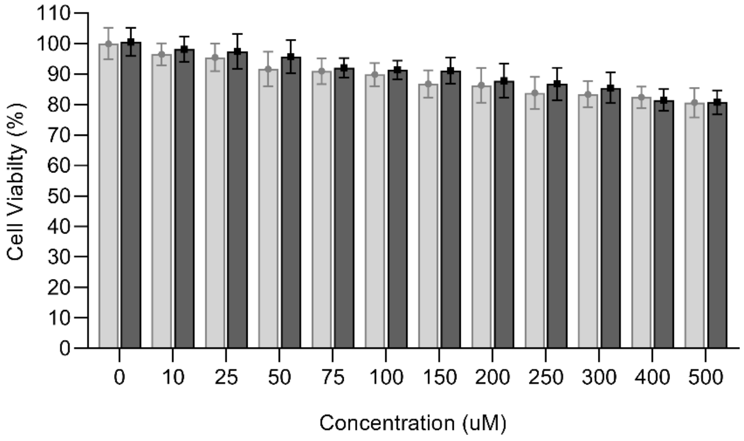

Figure S14. ***In vitro*** **cytotoxicity of PBP-NPs in AML12 cells.** Dose-dependent cell viability of PBP-NPs were evaluated by MTT assay in hepatocytes (n=3). Data are presented as means ± SD.

`

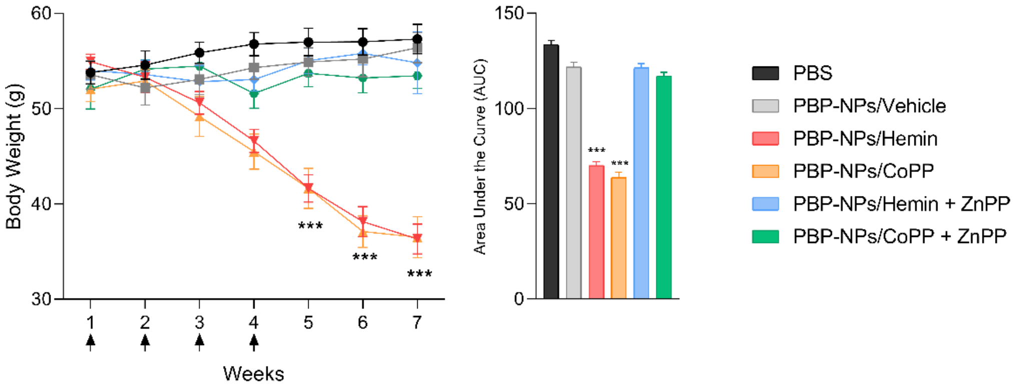

Figure S15. Anti-obesity effects of PBP-NPs in the high-fat high-fructose diet-induced nonalcoholic steatohepatitis mouse model. Measurements of body weight change. The high-fat high-fructose diet-induced nonalcoholic steatohepatitis mouse model was intravenously injected with 1 mg/kg of nanoparticles once a week for 4 weeks and ZnPP, an HO-1 inhibitor, was intraperitoneally injected to study competitive inhibition with PBP-NPs containing HO-1 inducers (n=5). Data are presented as means ± SD. ns = not significant, *p < 0.033, **p < 0.01, ***p < 0.001, by one-way ANOVA with Tukey's post hoc test were considered.

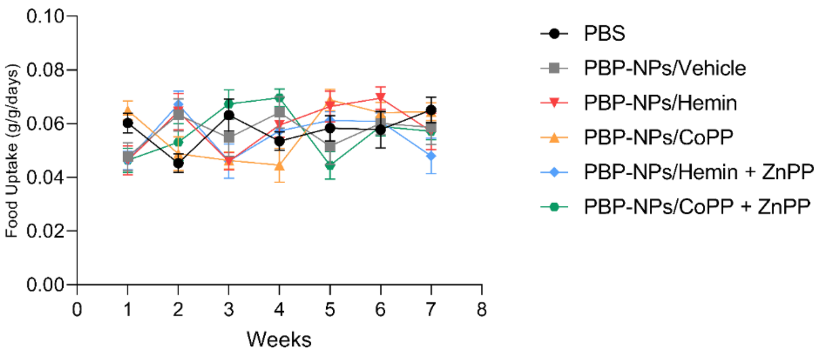

Figure S16. Patterns of weekly food uptake in the NASH model after treatments. A high-fat high-fructose diet-induced nonalcoholic steatohepatitis mouse model was treated with PBP-NPs for 4 weeks and observed additional 3 weeks. Amounts of HFD were measured every other day and normalized with the sum of the body weights of each group (n=3). Data are presented as means ± SD.

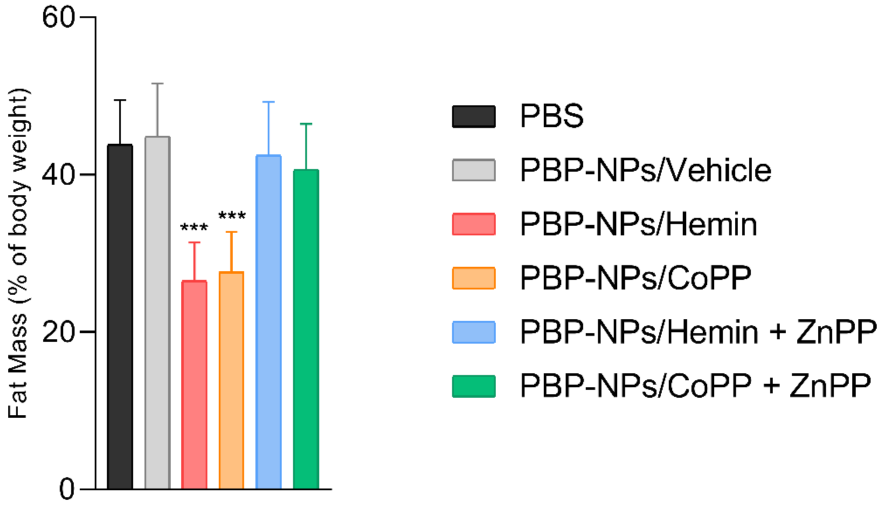

Figure S17. Measurement of total fat mass in high-fat high-fructose diet-induced nonalcoholic steatohepatitis mouse model. A high-fat high-fructose diet-induced nonalcoholic steatohepatitis mouse model was treated with PBP-NPs for 4 weeks and observed additional 3 weeks. Amounts of total fat tissue were measured and normalized with body weights (n=5). Data are presented as means ± SD. ns = not significant, *p < 0.033, **p < 0.01, ***p < 0.001, by one-way ANOVA with Tukey's post hoc test were considered.

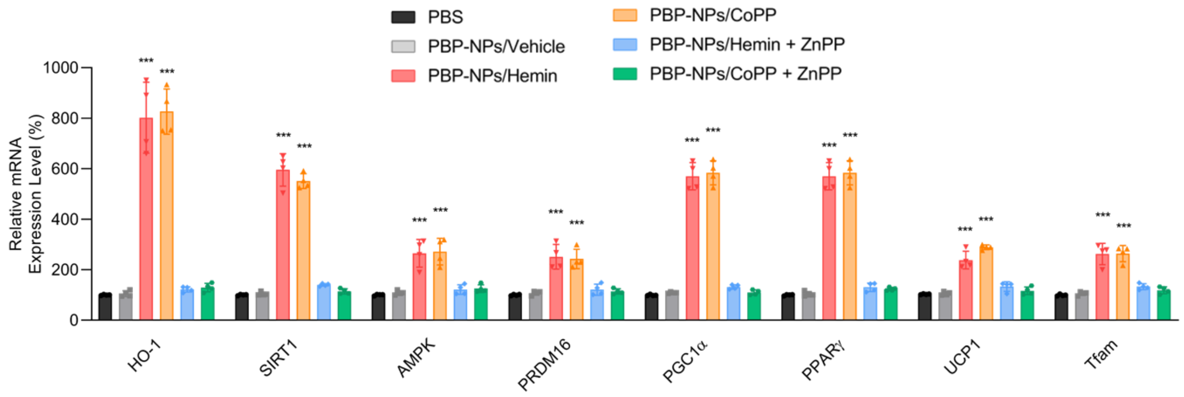

Figure S18. Effects of brown adipogenesis by PBP-NPs in visceral adipose tissue of high-fat high-fructose diet-induced nonalcoholic steatohepatitis mouse model. Relative mRNA expression levels of HO-1, downstream signal (SIRT1 and AMPK), brown adipogenesis marker (PRDM16, PPARγ, and PGC1α), and mitochondrial biogenesis marker (UCP1 and Tfam) in visceral adipose tissue. The mRNA levels were normalized to that of GAPDH mRNA by qPCR (n=4)**.** Data are presented as means ± SD. ns = not significant, *$p < 0.033$, **$p < 0.01$, ***$p < 0.001$, by one-way ANOVA with Tukey's post hoc test were considered.

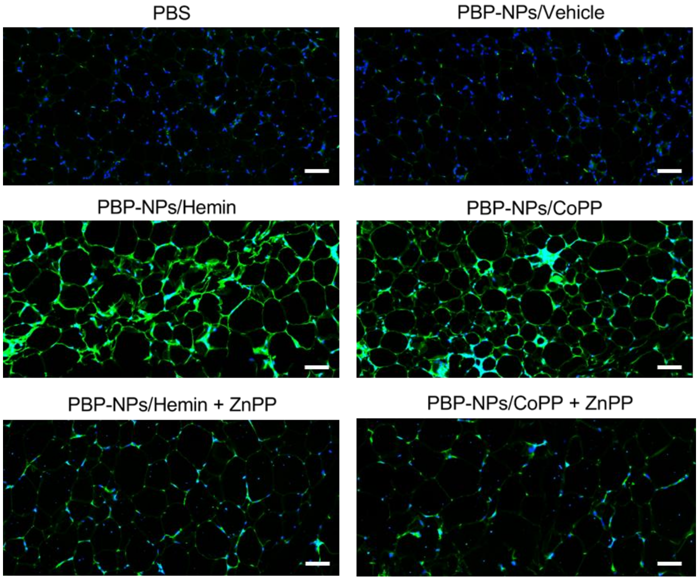

Figure S19. Effects of UCP1 upregulation by PBP-NPs in visceral adipose tissue of high-fat high-fructose diet-induced nonalcoholic steatohepatitis mouse model. Immunofluorescence staining of UCP1 (green) in visceral adipose tissue embedded in paraffin. Section thickness = 6μm. Scale bar = 200μm.

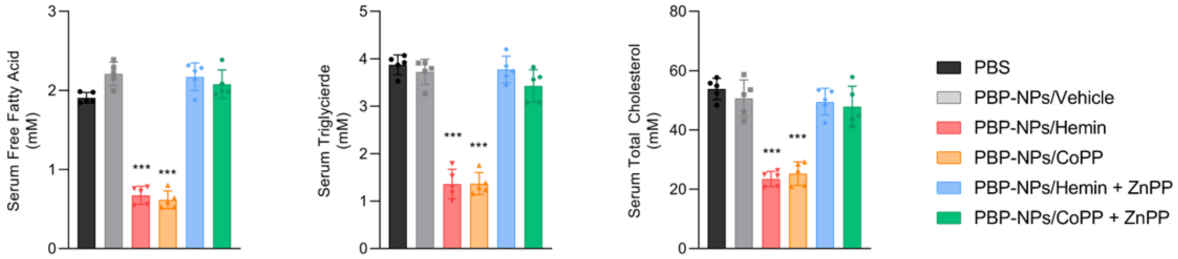

Figure S20. Reversal of hyperlipidemia by PBP-NPs in the high-fat high-fructose diet-induced nonalcoholic steatohepatitis mouse model. Free fatty acids, triglyceride, and total cholesterol levels in serum were measured (n=5). Data are presented as means ± SD.

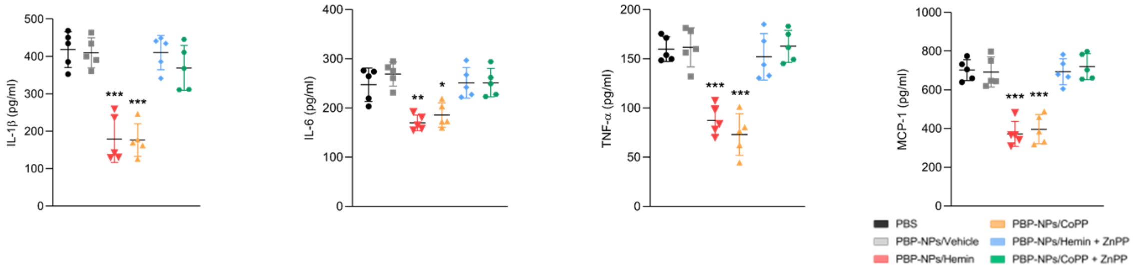

Figure S21. Reduction of inflammatory cytokines by PBP-NPs in visceral adipose tissue of the high-fat high-fructose diet-induced nonalcoholic steatohepatitis mouse model. Inflammatory cytokines from visceral adipose tissue were analyzed by ELISA (n=5). Data are presented as means ± SD. ns = not significant, *p < 0.033, **p < 0.01, ***p < 0.001, by one-way ANOVA with Tukey's post hoc test were considered.

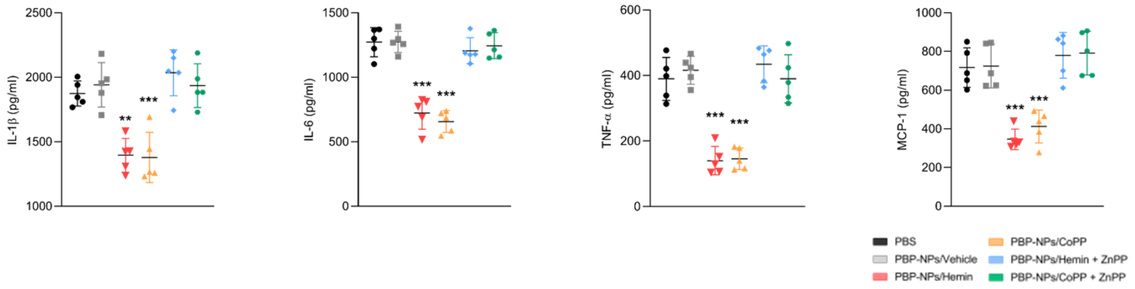

Figure S22. Reduction of inflammatory cytokines by PBP-NPs in the serum of the high-fat high-fructose diet-induced nonalcoholic steatohepatitis mouse model. Inflammatory cytokines from serum were analyzed by ELISA (n=5). Data are presented as means ± SD. ns = not significant, *p < 0.033, **p < 0.01, ***p < 0.001, by one-way ANOVA with Tukey's post hoc test were considered.

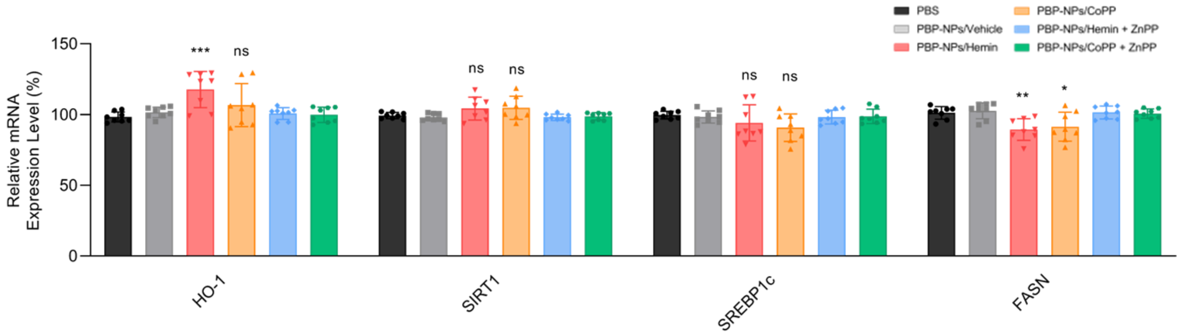

Figure S23. mRNA expression in liver of high-fat diet-induced type 2 diabetic mouse model. a) Relative mRNA expression levels of HO-1, SIRT1, lipid metabolism marker (SREBP1c and FASN) in fatty liver. The mRNA levels were normalized to that of GAPDH mRNA by qPCR (n=8). Data are presented as means ± SD. Data are presented as means ± SD. ns = not significant, *p < 0.033, **p < 0.01, ***p < 0.001, by one-way ANOVA with Tukey's post hoc test were considered.
